# Eukaryotic and Prokaryotic Microbiota Interactions

**DOI:** 10.3390/microorganisms8122018

**Published:** 2020-12-17

**Authors:** Aly Kodio, Estelle Menu, Stéphane Ranque

**Affiliations:** 1 Aix-Marseille Université, Instiut de Recherche pour le Développement, Assistance Publique Hôpitaux de Marseille, Service de Santé des Armées, Vecteurs et Infections TROpicales et MEditerranéennes, 13005 Marseille, France; alykodio81@gmail.com (A.K.); menu.estelle1@gmail.com (E.M.); 2 IHU-Méditerranée Infection, 13005 Marseille, France; 3 Malaria Research and Training Center, International Centers for Excellence in Research, Université des Sciences, des Techniques et des Technologies de Bamako, Bamako BP 1805, Mali

**Keywords:** microbiota, mycobiota, interactions, host, NGS, metagenomics, culturomics, metabarcoding

## Abstract

The nature of the relationship between the communities of microorganisms making up the microbiota in and on a host body has been increasingly explored in recent years. Microorganisms, including bacteria, archaea, viruses, parasites and fungi, have often long co-evolved with their hosts. In human, the structure and diversity of microbiota vary according to the host’s immunity, diet, environment, age, physiological and metabolic status, medical practices (e.g., antibiotic treatment), climate, season and host genetics. The recent advent of next generation sequencing (NGS) technologies enhanced observational capacities and allowed for a better understanding of the relationship between distinct microorganisms within microbiota. The interaction between the host and their microbiota has become a field of research into microorganisms with therapeutic and preventive interest for public health applications. This review aims at assessing the current knowledge on interactions between prokaryotic and eukaryotic communities. After a brief description of the metagenomic methods used in the studies were analysed, we summarise the findings of available publications describing the interaction between the bacterial communities and protozoa, helminths and fungi, either in vitro, in experimental models, or in humans. Overall, we observed the existence of a beneficial effect in situations where some microorganisms can improve the health status of the host, while the presence of other microorganisms has been associated with pathologies, resulting in an adverse effect on human health.

## 1. Introduction

Several microorganisms have been isolated from different body parts of living beings. The community of microorganism living within a body, referred to as “microbiota”, is made up of bacteria, archaea, fungi, protozoa, metazoans (mainly helminths) and viruses. Viruses, including giant viruses, have been found to be part of microbiota [1,2,3]. The microbiota varies from one human to another and its composition and diversity may be influenced by interactions between host genetics, immune response, diet and the physiological and pathological [4] conditions of the environment [5]. Other factors potentially influencing the bacterial intestinal microbiome include diet (animal proteins, fatty acids, carbohydrates, processed foods, dietary fibres) [6,7,8], age [5], stool consistency [9,10], physiological and metabolic status [11,12], medical practices (e.g., antibiotic treatments) [13], seasons [14] climate change [15], seasonal cycles [14] and the host’s genetic background [12,16,17,18]. The core human microbiota is composed of at least 1800 genera and up to 40,000 bacterial strains [19] that carry around 10 million non-human genes [20]. Studies of human microbiota were carried out by Antonie van Leeuwenhoek at the end of the 17th century following his discovery of “animalcules” through the microscopic observation of human mouth scrapings [21]. The microbiological application of DNA-based assays, nucleotide sequencing and matrix-assisted laser desorption ionisation time-of-flight mass spectrometry (MALDI-TOF-MS) considerably enhanced capacities to identify microorganisms [22,23].

The opportunistic and pathogenic nature of various microorganisms is increasingly reported in Inflammatory Bowel Disease therapy, fostered by the growing use of immunosuppressive therapies [24]. To date, several studies have led to an enlargement of the bacterial microbiota repertoire which is critical to establishing the link between diseases and the bacterial species involved [25,26]. While there are many studies on the isolation, identification and phenotypic and genomic characterisation of prokaryotic communities, few studies have aimed at describing eukaryotic communities and their influence on the bacterial microbiota. Studies have shown that *Blastocystis* protozoa are associated with a healthy digestive microbiota [14,27,28,29] while protozoa such as *Giardia duodenalis* [28,30], and *Entamoeba histolytica* [31] have been linked to dysbiosis situations. In addition, studies have suggested that the gut bacterial community structure is associated with a risk of *P. falciparum* infection [32] and also of severe malaria [33]. The addition of *Lactobacillus* and *Bifidobacterium* probiotics to the gut microbiota has been advocated to reduce *Plasmodium* parasite density [33]. A study reported that *Enterobius vermicularis* infection in children was associated with an increased gut microbial diversity, a higher relative abundance of *Alistipes* and *Faecalibacterium*, whereas the quantity of Acidaminococcus, Megasphaera, Veillonella and Fusobacterium was relatively low compared to the non-infected group [34]. A lower diversity of mouse gut microbiota and an increase in the relative abundance of *Lactobacillaceae* has been observed following a *Trichuris muris* infection [35]. The results of another study pointed out that Nematode infection triggers both qualitative and quantitative changes in the microbiota that can significantly alter the microbial metabolism and thus influence the host’s nutrition and immunity [36]. It has been suggested that interactions between bacterial and fungal communities are involved in *Clostridium difficile* infection pathophysiology and in the persistence and recurrence of *Clostridium difficile* infections [37]. Moreover, a study comparing fungal microbiota in the guts of healthy subjects and patients with Crohn’s disease highlighted a fungal community dysbiosis in the Crohn’s disease cohort, suggesting that fungi may be involved in the pathogenesis of inflammatory bowel diseases [38]. While studies on the eukaryotic microbiota remain relatively scarce, this review aimed at assessing the current knowledge on interactions between the prokaryotic and eukaryotic communities, derived from studies based on microbial genomics, metagenomics and/or culturomics approaches.

## 2. Methods

### 2.1. Literature Search Strategy

We searched for articles in PubMed and Web of Science. We used the following Mesh terms in PubMed by limiting articles to those on the microbiota (human, animal) and by publication date (20 March 2008 to 3 February 2020): (“parasites” [mesh Major Topic] or “protozoa” [All Fields] or “protozoan infections/parasitology” [Mesh terms] or “helminths” [Mesh major topic] or “helminthiasis/drug therapy” [Mesh terms] or “fungi” [Mesh major topic] or “mycobiome” [Mesh terms]) and (“Bacteria/microbiology” [Mesh major topic] OR “gastrointestinal microbiome” [Mesh terms]) and (“20 March 2008” [ PDat]: “3 February 2020” [PDat] AND (“humans” [Mesh terms] OR “animals” [Mesh terms: noexp])). The search for articles on the Web of Science was carried out according to the keywords as follows: ((microbiota AND parasites AND host parasite interactions) OR protozoan infections AND metagenomics OR gastrointestinal microbiome)). The results of the search were refined by category and by date: (microbiology OR parasitology) AND publication years: (2016 OR 2011 OR 2017 OR 2009 OR 2015 OR 2008 OR 2012 OR 2018 OR 2013 OR 2014 OR 2010). We excluded these categories of research: (biochemistry molecular biology OR virology OR immunology OR cell biology OR dentistry oral surgery medicine OR marine freshwater biology OR ecology OR public environmental occupational health) and [excluding] Web of Science categories: (food science technology OR pharmacology pharmacy OR biochemical research methods).

### 2.2. Data Extraction

**Figure 1:**
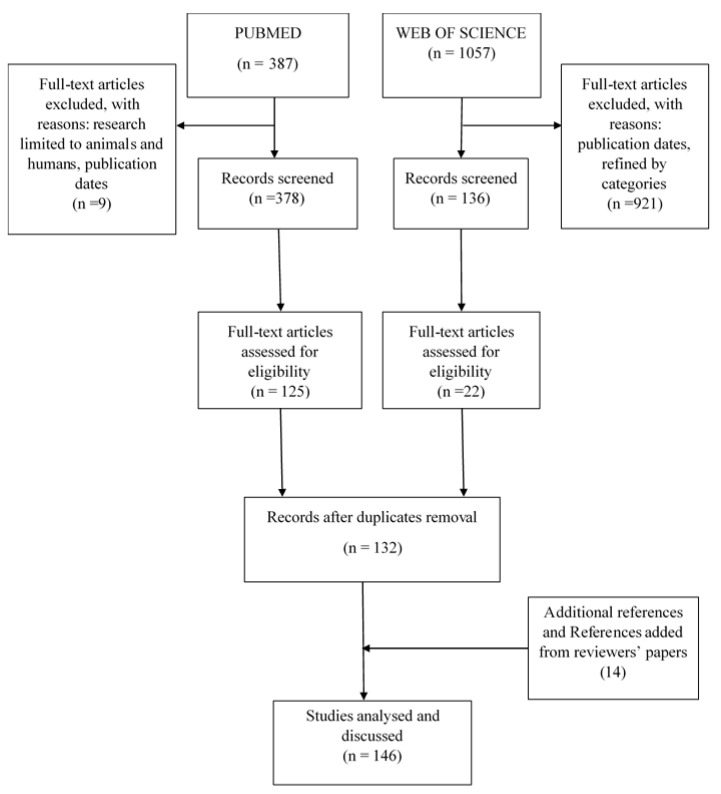
Flow diagram of the selection of articles and data extraction.

The search for articles corresponding to the MeSH terms used in the PubMed search database resulted in 387 articles. By focusing on animal and human microbiota and limiting research to the publication range to between 20 March 2008 and 3 February 2020, 378 articles were retained. The Web of Science search database generated 1057 articles using the keywords mentioned above. By limiting the research to the domain of microbiology and parasitology and to the year of publication (2008–2018), 136 articles were retained. The evaluation of the title and text of 378 PubMed articles and 136 Web of Science articles involving the bacterial community in relation to protozoa, helminths and fungi resulted in 125 and 22 articles, respectively. The introduction of eligible articles by both search engines into the bibliographic database, Zotero (www.zotero.org), eliminated duplicates and 132 articles were retained. Considering reviews of the relationships between bacterial and protozoa, helminths, or fungal communities, 14 articles of interest were added to the bibliographic database and 146 articles were included for qualitative synthesis (see Figure 1 for details).

## 3. Results

### 3.1. Different Methods Characterizing Microbiota

In this preliminary section, we will briefly describe the methods that were used to characterise the microbial communities in the articles analysed including: culturomics and culture-based methods; polymerase chain reaction; denaturing gradient gel electrophoresis (DGGE); high throughput sequencing of 16S rRNA amplicons and whole genome shotgun (WGS).

#### 3.1.1. Culturomics and Culture-Based Methods

Culture-based methods are traditionally used for isolating bacteria from the digestive tract. The majority of bacteria cannot be cultivated in the conventional laboratory environment, resulting in an underestimation of the actual richness of species and an overestimation of the importance of the species that grow disproportionately well under standard laboratory conditions [39,40]. Culture has enabled the development of new knowledge, which is important in the identification of antibiotic resistance, the study of virulence, genomic sequencing of microorganisms and the detection of organisms in low abundance, such as *Clostridium difficile*, by using selective growth media [22,40]. “Culturomics” refers to the intensive culture of bacteria using different types of media and conditions (i.e., aerobic and anaerobic) and often adding nutrients to the culture media to mimic natural habitat conditions. Culturomics has contributed significantly to the discovery of new bacterial species [41].

#### 3.1.2. Polymerase Chain Reaction

Quantitative real time PCR (qPCR) assays are commonly used to detect specific microorganisms. This tool can be used to detect all the members of a given taxon present in a sample, thus estimating the abundance of this taxon within the studied microbiota. qPCR requires primers and probes that are specific to a given taxon and allows quantification of the amplicons. However, it is prone to PCR biases, including amplification errors, formation of chimeric and heteroduplex molecules and preferential amplification. Increasing the resolution requires a set of primers with different specific probes to target each taxon which can be amplified either within the same reaction (known as “multiplexing”), or separately. Multiplexing further complexifies the procedure and increases the time and cost required to obtain a relatively limited amounts of data [40].

#### 3.1.3. Denaturing and Temperature Gradient Gel Electrophoresis (DGGE)

Denaturing and temperature gradient gel electrophoresis (DGGE) consists of separating DNA fragments on an electrophoresis gel containing a denaturing agent (such as urea, formamide) according to their physical-chemical properties into a series of bands whose characteristics can be compared between communities [40]. The DGGE method has been widely used for the study of bacterial genetic diversity [42]. However, it is limited by the lack of taxonomic resolution. It makes it possible to compare different community structures but cannot identify the taxa accounting for that difference [40]. The DGGE method has the advantage, at a lower cost, of rapidly developing an image of the diversity and structure of microbial communities from several environmental samples. It has been used in the analysis of complex communities [43], in monitoring population dynamics [44], in the detection of sequence heterogeneities [45], in the comparison of DNA extraction methods yields [46], in the detection of clone banks [47] and in the determination of PCR and cloning biases [42,48]. DGGE makes it possible to cut, re-amplify and sequence the bands to obtain taxonomic information [40,48,49].

DGGE assays are limited by the heterogeneous effectiveness of DNA extraction procedures [50], PCR biases (see before) [51] and potential contamination during DNA and PCR extraction [42]. It has been noted that only fragments below 500 bp can be separated by DGGE, thus limiting sequence information. Furthermore, interpretation can sometimes be difficult as bands at similar positions do not necessarily correspond to identical sequences but may be sequences which share the same melting behaviour [52].

#### 3.1.4. Sequencing of 16S rRNA Amplicons or 16S Metabarcoding

The 16S rRNA metabarcoding method is the most widely used method to analyse the bacterial microbiome. It uses the 16S rRNA gene barcode for taxonomic classification, which contains highly conserved regions, present in the majority of bacterial genomes and hypervariable regions that allow taxa to be discriminated [40]. The sequencing of the 16S rRNA gene is simplified and provides a high depth of taxonomic resolution [53].

The Sanger sequencing method can perform reads with lengths of more than ~1000 bp and a raw accuracy per base of up to 99.99%. High throughput shotgun Sanger genomic sequencing is useful for small projects from kilobase to megabase, and the technology is likely to be excellent within a short period of time [54]. Second generation DNA sequencing, using alternative DNA sequencing strategies, has been categorised using micro electrophoretic methods, hybridisation sequencing, real-time observation of single molecules and cyclic network sequencing [55,56,57,58,59]. Below, we list some technologies commercialised in the cyclic network sequencing category (e.g., 454 (used in the 454 Genome Sequencer, Roche Applied Science; Basel), Solexa Technology (used in the Illumina Genome Analyser (San Diego, CA, USA)), SOLiD platform (Applied Biosystems; Foster City, CA, USA), Polonator (Dover/Harvard) and the HeliScope Single Technology Molecule Sequencer (Helicos; Cambridge, MA, USA) [54]. Historically, as the first-generation sequencing commercially available system, the 454 Roche pyrosequencer provided long read lengths to obtain a highly informative 16S RNA fraction [60]. The second-generation using cyclic-array strategies has many advantages over Sanger sequencing, and can be summarised as follows: (1) the generation of sequencing characteristics obtained following the construction of a sequencing library, followed by in vitro clonal amplification, allows several choke points to be overcome; (2) sequencing through cyclic-array strategies provides a higher degree of parallelism than conventional capillary-based sequencing; (3) the cost of DNA sequence production can be reduced by adjusting the volume of the reagent from microlitres to femtolitres when immobilising array elements on a planar surface that can be processed by a single volume. Second-generation sequencing has, however, some disadvantages, including the length of the reads and raw precision which is lower when compared to Sanger sequencing [54].

Recently, sequencing technology has evolved to bench-top sequencers within the reach of small laboratories, namely the 454 GS Junior, the Ion Torrent Personal Genome Machine (PGM) and Proton and the Illumina MiSeq and NextSeq 500. These bench-top sequencers offer multiple advantages over large-scale sequencers. They can provide fewer reads per run and fewer bases per dollar. They are more adaptable, faster and their low acquisition and operating costs make them affordable. The benchtop next-generation sequencers are described as being better suited for environmental microbiology studies given the generation of large amounts of sequence data with maximum yields of ~35 Mbp (454 GS Junior), ~2 Gbp (PGM Ion Torrent), ~10–15 Gbp (Ion Torrent Proton), ~10 Gbp (Illumina MiSeq) and ~100 Gbp (Illumina Next Seq 500) [53].

#### 3.1.5. Whole Genome Shotgun (WGS)

**Figure 2:**
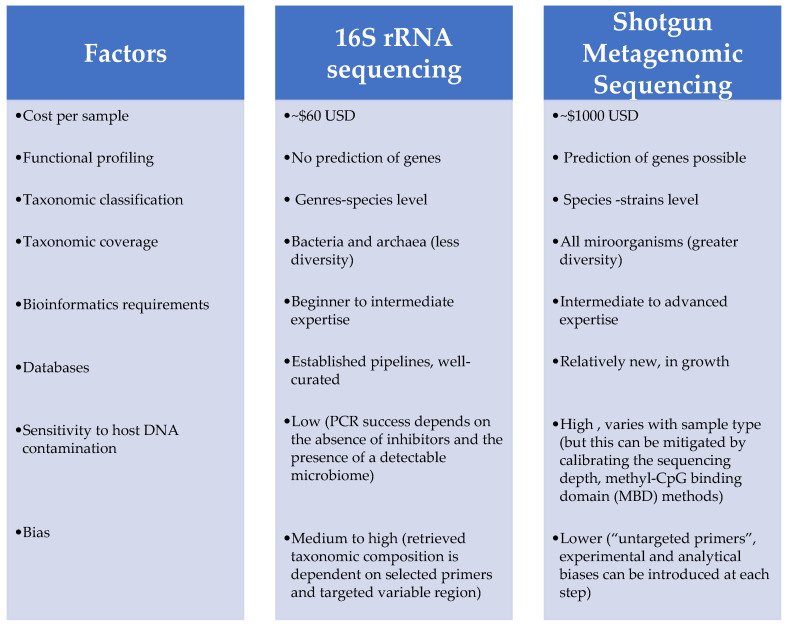
Summary of some differences between the 16S RNA sequencing methods and the metagenomic sequencing shutgun [65,66,67,68,69,70,71,72].

Significant advantages of the WGS method have been reported in bacterial microbiome analysis studies. The WGS method has been suggested as an alternative to the 16S RNA sequencing method, which is a method used to sequence random DNA strands. The main advantages of the WGS method are that taxa can be more precisely defined at the species level. It is worth noting that 16S RNA sequencing and the WGS method use distinct databases for taxa classification [61,62,63,64]. Sequencing the entire genome of the shotgun has multiple advantages over the 16S amplicon method, including a higher sensitivity in the detection of bacterial species, an increase in the detection of diversity and an increase in the prediction of genes. In addition, the increase in nucleotide sequence length, due either to long reads or to the assembly of contigs, has considerably improved the accuracy of species detection. Nevertheless, WGS is more expensive than the 16S rRNA amplicon sequencing method and requires more in-depth data analysis (Figure 2) [65]. It may also be necessary to sequence a high-coverage genome in order to identify and understand the genes of a bacterial taxon.

### 3.2. Impact of Eukaryotes on Bacterial Community

After this brief introduction to the methods that were used to characterise the microorganism communities in the studies we analysed, we will further detail the (i) in vitro, (ii) experimental and (iii) clinical data that are available on the interaction between bacterial community and (1) protozoa, (2) helminth and (3) fungi and also their impact on bacterial community diversity.

#### 3.2.1. Protozoa—Bacterial Community Interaction

##### Impact of Protozoa on Bacterial Community Diversity

In this section, we describe the influence of protozoa (*Cryptosporidium parvum*, *Giardia* sp., *Blastocystis* sp., *Entamoeba* sp., *Plasmodium yoelii*, *Leishmania infantum*, *Toxoplasma gondii*, *Trichomonas vaginalis*, *Cystoisospora*) on bacterial diversity in humans and animals.

The presence of *Cryptosporidium parvum* has been described as upsetting the native intestinal microbiota in mice, with a taxonomic analysis showing an increased abundance of the phylum unclassified *Bacteroidetes*, *Porphyromonadaceae* and *Prevotellaceae* in the infected groups [73]. The presence of *Giardia* sp. has been associated with various changes in microbiota diversity. Several studies relating to the faecal microbiota in animal models or human cohorts have reported an increased abundance of the phylum *Firmicutes* among infected subjects [28,30]. A highly heterogeneous description of the diversity of gut microbiota during *Blastocystis* sp. infection has been reported in various studies, although they all seem to conclude in favour of a beneficial impact on the gut microbiota [28,74]. Divergences have been observed regarding the relative abundance of some species (e.g., *Faecalibacterium prausnitzii*, *Prevotella*); however, some associations such as the negative association between *Blastocystis* spp. and *Bacteroides* in stool samples have been consistently reported [27,28,29,74]. *Entamoeba* spp. infection has been shown to disturb the bacterial microbiota by increasing its diversity [75]. However, given the variability of the tools used to study the microbiota, there are discrepancies regarding the abundance of some bacteria (e.g., *Prevotella copri* during *Entamoeba* spp. infection) [31,75]. Other protozoa play a role in modulating the microbiota of bacteria. Thus, a reduction in the diversity of the microbiota has been described during infection with *Plasmodium yoelii* [33] and *Leishmania infantum* [76]. The abundance of some bacterial genus of medical interest, such as *Lactobacillus*, increases during *Toxoplasma gondii* infections [33,77] and decreases during *Trichomonas vaginalis* infections [78]. Similarly, *Bifidobacterium* abundance increases during *Cystoisospora* infections in cats [79] and decreases during *Toxoplasma gondii* infections in mice [77]. A study has shown that *Lactobacillus* and *Bifidobacterium*, when used as a probiotic in mice infected with *Plasmodium yoelii*, resulted in decreased *Plasmodium* load [33].

##### Impact of Protozoa on Bacterial Community Structure

In this section, we will analyse the interactions between protozoa and the bacterial communities, in vitro and in vivo, from experimental or clinical studies (Table 1). We describe the impact of *Giardia* spp., *Cryptosporidium parvum*, *Toxoplasma gondii*, *Plasmodium* spp., *Leishmania infantum*, *Cystoisospora* spp., *Blastocystis* spp., *Entamoeba* spp., *Dientamoeba fragilis* and *Trichomonas vaginalis* on the gut microbial community in vitro, in animals and in humans.

(a) In vitro studies

An in vitro study concerning the interaction between *Giardia intestinalis* and different lactobacilli demonstrated that both the *Lactobacillus acidophilus* NCC 2628 strain isolated from dog faeces and the probiotic *Lactobacillus johnsonii* La1 significantly inhibited the proliferation of *G. intestinalis* trophozoites [84].

(b) Experimental studies

In experimental studies, Barash et al. used cultivation-independent methods to evaluate microbial diversity and the impact of *Giardia* infection on the gut microbiota by infecting mice with *Giardia lamblia*. In this study, infection with *Giardia lamblia* was associated with an increase in *Proteobacteria* diversity and a decrease in *Firmicutes* and *Melainabacteria* diversity in the foregut and hindgut [82]. The authors showed that the microbial structure due to *Giardia* associated-dysbiosis differed depending on the region of the gut. Thus, during giardiasis, the relative abundance of *Rhodocylaceae* increased in the proximal small intestine, while an enrichment of *Moraxellaceae*, *Flavobacteriales*, *Comononadaceae* and *Bacteroidales* was observed throughout the small intestine, and *Clostridiacae* were depleted across the intestinal tract [82]. In contrast, germ-free mice who received *Giardia*-infected microbiota showed an increase in *Firmicutes*, associated with a decrease in *Phascolarctobacterium* [30]. The study of the disturbance of the faecal bacterial microbiota of mice infected with *Cryptosporidum parvum* by metabarcoding analysis showed that *Bacteroidetes*, *Prevotellaceae* and *Porphyromonadaceae* unclassified OTUs were over-represented in *C. parvum* infected mice, whereas distinct *Porphyromonadaceae* and unclassified *Bacteroidetes* OTUs were over-represented in the non-infected mice [73]. Another study showed that severe *Cryptosporidium parvum* infection in mice was associated with an increased abundance of *Proteobacteria* and decreased abundance of *Firmicutes* [81]. Regarding changes to the intestinal microbiota during *Toxoplasma gondii* ileitis, both metagenomic and quantitative PCR analyses of the intestinal bacterial microbiota in NOD2^−/−^ mice and C57BL/6 wild type mice showed an increase in *Enterobacteria*, *Enterococci* and *Bacteroidetes*/*Prevotella* species during *T. gondii* ileitis. In particular, the total eubacterial load increased only in NOD2^−/−^ mice [77]. Furthermore, *Toxoplasma gondii* infection in mice led to an overgrowth of *Clostridia* spp. within the gut microbiota during the chronic stage of the disease, unrelated to the symptomatology [83]. Regarding *Plasmodium* infection, Villarino et al. [33] used metagenomic analysis to show that the abundance of *Clostridiaceae*, *Erysipelotrichaceae*, *Lactobacillaceae* and *Peptostreptococcaceae* increased in resistant (Jax and Tac) mice to *Plasmodium yoelii*, whereas the abundance of *Bacteroidaceae*, *Prevotellaceae* and *Sutterellaceae* increased in susceptible (NCI and Har) mice. In addition, these authors showed that the abundance of *Lactobacillus* and *Bifidobacterium* increased in mice resistant to *Plasmodium yoelii* and their use as probiotics decreased parasitic load. The study of *Plasmodium chabaudi* infection in mice showed an enhanced intestinal bacterial translocation during *Plasmoduim* infection, promoting non-typhoidal *Salmonella* bacterial dissemination from the intestinal tract [85].

In animal study, V4 region 16S metabarcoding with the Ion Torrent PGM^™^ platform showed an increase in *Catenibacterium*, *Pseudomonas* and *Howardella* and a decrease in *Bacteroides* and *Pseudobutyrivibrio* following *Giardia duodenalis* infection in the gut bacterial communities of healthy dogs. The study of the structure and composition of gut microbiomes from healthy dogs and cats with or without *Giardia* infection and coccidia demonstrated an increase in *Roseburia* and a decrease in the abundance of *Subdoligranulum* following *Giardia cati* infection in cats. An increase in *Bifidobacterium*, *Olsenella*, *Megamonas*, *Geobacillus*, *Meiothermus*, *Bacillus*, *Camonas*, *Schlegelella*, *Chelatococcus* and *Silanimonas* was also associated with the presence of *Cystoisospora* in cats [79].

Regarding *Leishmania infantum*, the metabarcoding analysis of the bacterial community within the midgut of one of its vectors, the sand fly *Lutzomyia longipalpis*, showed a progressive decrease in bacterial richness and *Pseudomonadaceae* abundance, whereas the abundance of *Acetobacteraceae* progressively increased following infection. The results of microbial community Fisher’s linear discriminant analysis (LDA) showed that members of the *Actinobacteria* phylum (e.g., *Tsukamurella*, *Tsukamurellaceae*, *Coprococcus*, *Porphyromonadaceae*, *Kocuria*, *Pigmentiphaga*) were predominant in sand flies infected by *L. infantum* [76]. In humans with *Leishmania donovani* complex associated with visceral leishmaniasis, 16S metabarcoding showed that *Ruminococcaceae* UCG-014 and Gastranaerophilales_uncultured bacterium were less abundant than in controls, and 18S rRNA metabarcoding showed an increase in *Pentatrichomonas* sp. and a decrease in *Entamoeba* sp. compared to controls. In the same subjects, a higher *Blastocystis* abundance was associated with a high bacterial diversity and a relatively low *Escherichia*-*Shigella* abundance. In addition, high *Blastocystis* abundance was associated with a relatively low Bacteroidaceae and high Clostridiales vadin BB60 abundance [86].

(c) Clinical studies

In one clinical study of *Blastocystis* spp. and intestinal bacterial microbiota interactions in cirrhotic patients with or without hepatic encephalopathy, 16S metabarcoding found a relatively high abundance of *Alkaliphilus* and *Flavobacterium* populations and a relatively low abundance of *Veillonella* and *Streptococcus* populations in *Blastocystis*-positive patients without hepatic encephalopathy [74]. Another 16S metabarcoding study on the impact of *Blastocystis* colonisation on the diversity of human gut bacterial microbiota found an increase in the relative abundance of the genera *Acetanaerobacterium*, *Acetivibrio*, *Coprococcus*, *Hespellia*, *Oscillibacter*, *Papillibacter*, *Sporobacter* and *Ruminococcus* in patients colonised by *Blastocystis* spp. than in *Blastocystis*-free patients. At the class level, this study reported that *Clostridia* abundance increased, whereas *Enterobacteriaceae* decreased in patients with *Blastocystis* spp. [29]. A study carried out in Malian children colonised by *Blastocystis* has shown similar results with higher microbiota diversity and more abundant beneficial bacteria. The phyla *Firmicutes*, *Elusimicrobia*, *Lentisphaerae*, *Euryarchaeota* and the species of *Faecalibacterium prausnitzii* (family *Ruminococcaceae*) and *Roseburia* sp. (family *Lachnospiraceae*) were associated with *Blastocystis* colonisation [80]. In terms of *Entamoeba* spp., a prospective cohort study of clinical enteric infections used qPCR detection to reveal a significantly higher level of *Prevotella. copri* in infants with diarrhoea due to *Entamoeba histolytica,* whereas the level of *Bacteroides thetaiotaomicron* was standard [31]. The presence of *Entamoeba dispar* and/or *E. histolytica* was associated with a decrease in the relative abundance of *Prevotella copri*, an increase in *Clostridiales Christensenellaceae*, *Elusimicrobiales Elusimicrobiaceae* and *Spirochaetaceae Treponema*, by 16S metabarcoding of the digestive microbiota in Pygmy hunter-gatherers and in the Bantu in Cameroon. *Clostridiales* and *Ruminococcaceae* displayed a significantly greater abundance in individuals with *Entamoeba* spp. [75]. Some studies analysed the interaction between the simultaneous presence of multiple protozoa and the bacterial microbiota. One study, aiming to assess the association between *Blastocystis* spp., *Dientamoeba fragilis* and intestinal bacteria, used qPCR and found a relative abundance of *Bacteroides* which was significantly higher in *Blastocystis* spp. and *Dientamoeba fragilis* negative groups compared to groups with the least one of these protozoa positive groups. The relative abundance of *Clostridial* cluster IV was significantly lower in the *Blastocystis*-positive/*Dientamoeba*-negative group compared with the *Blastocystis*-negative/*Dientamoeba*-positive group and the relative abundance of *Clostridial* cluster XIVa was higher in the *Blastocystis*-negative/*Dientamoeba*-negative group compared with the *Blastocystis*-positive/*Dientamoeba*-negative group [27]. By studying the impact of *Giardia duodenalis*, *Entamoeba* spp. and *Blastocystis hominis* infections on the human gut microbiota using qPCR analysis, Iebba et al. found that *Giardia* spp. infection was associated with a dysbiotic condition explained by a slight increase in *Escherichia coli* levels and increase in *Bifidobacterium*. Furthermore, *Entamoeba* spp./*Blastocystis hominis* were associated with a eubiotic condition described as a significantly higher *Faecalibacterium prausnitzii*-*Escherichia coli* ratio in the faecal bacterial community in people from the Ivory Coast [28].

The impact of protozoa on the composition and structure of the vaginal microbiota has also been studied using a similar approach. The relationship between vaginal bacterial community and *Trichomonas vaginalis* infection showed a decreased abundance in *Lactobacilli* and an increased abundance of *Mycoplasma*, *Parvimonas* and *Sneathia* [78].

#### 3.2.2. Interaction of Helminths and Bacterial Community

##### Influence of Helminths on Bacterial Community Diversity

According to experimental studies, *Trichiuris* spp. mono-infection has no impact on the diversity of the gut microbiota in the porcine colon [87,88], but, when it comes to mixed infections, the data diverge: one study reported reduced bacterial diversity in children with a mixed infection involving *T. trichiura* and *Ascaris lumbricoides* [89], whereas others showed an increase in bacterial diversity among helminth-infected adults and children with *Trichuris*, hookworms and/or *Ascaris* [90,91]. *Trichiuris* spp. infection has been associated in humans with an increased abundance of the *Prevotella* genus [89,90] but a significant reduction in their proportions of the microbiota in mice infected with *T. muris* [92]. Likewise, clinical studies concerning the bacterial diversity of the gut microbiota report an enrichment of bacterial taxa among *Ascaris*-infected patients for some [91] and a reduced overall diversity during *Ascaris* spp. infection for others [89]; however, an experimental study on the analysis of the gut microbiota composition in pigs infected with *A. suum* showed a trend for increased microbial diversity [93]. A higher abundance of *Prevotella* has also been observed with the presence of *Ascaris* spp alone or in combination with other helminths [94,95]. In addition, *Necator americanus* infection has been associated with an increased in the species richness but not in the bacterial diversity in patients with celiac disease [96]. An increase in bacterial community diversity has also been found in mixed infections with *Leidynema appendiculatum*, *Hammerschmidtiella diesingi*, *Thelastoma bulhoesi* in both *Periplaneta fuliginosa* and *Periplaneta americana* cockroach species [97]. Similarly, bacterial community diversity was also higher in *Ovis aries* sheep infected by larval-stage *Haemonchus contortus* [98]; after *Enterobius vermicularis* infection [34] and *Schistosoma mansoni* and *Schistosoma haematobium* infections [99] in children. The high abundance of *Proteobacteria* and lower abundance of *Firmicutes* has been observed in mixed *Leidynema appendiculatum*, *Hammerschmidtiella diesingi* and *Thelastoma bulhoesi* infections in cockroaches [97]. *Schistosoma mansoni* and *Schistosoma haematobium* infections in children showed a high abundance of *Firmicutes* and *Proteobacteria* [99]. In addition, *Enterobius vermicularis* infection in children was associated with an increased abundance in *Bifidobacterium longum* and *Faecalibacterium prausnitzii* species and was associated with greater bacterial community diversity [34].

##### Impact of Helminths on Bacterial Community Structure

In this section, we will describe the influence of *Heligmosomoides polygyrus bakeri*, *Trichuris* spp., *Hymenolepsis diminuta*, *Trichostrongylus retortaeformis*, *Toxocara cati*, *Leidynema appendiculatum*; *Hammerschmidtiella diesingi*; *Thelastoma bulhoesi*, *Enterobius vermicularis*, *Ascaris lumbricoides*, *Necator americanus*, *Schistosoma haematobium* on bacterial community in experimental or clinical studies (Table 2). We found no in vitro studies aiming at dissecting the interaction between helminths and bacterial communities.

(a) Experimental studies

In experimental studies, *Heligmosomoides polygyrus bakeri* infection was associated with a significant increase in *Lactobacillaceae* abundance, using 16S rRNA Sanger sequencing and qPCR, in the ileum and with improved disease in an inflammatory bowel disease (IBD) mouse model [107]. In *Apodemus flavicollis*, 454-pyrosequencing 16S V1V3 metabarcoding showed that *Lachnospiraceae* abundance increased in *H. polygyrus* infected mice and decreased in *Syphacia* spp. infected mice. In addition, *Syphacia* infection was associated with a decrease in of Firmicutes (*Lactobacillus*) OTUs, as opposed to *H. polygyrus.* An increase in the unidentified bacteria belonging to the phylum of Bacteroides (S24-7 OTUs) was observed in mice infected by *Hymenolepis* spp [102]. In addition, γ-*Proteobacteria*/*Enterobacteriaceae* and abundance of the bacteria of the *Bacteroides*/*Prevotella* group in the caecum, assessed via qPCR, was increased 14 days after *Heligmosomoides polygyrus bakeri* infection in mice [101]. In an obese mouse model, *Heligmosomoides polygyrus* infection suppressed weight gain and increased the abundance of *Firmicutes* and *Proteobacteria* phyla, as was the case for *Bacillus* and *Escherichia* genera, in the gut bacterial community [108]. In *Schistosoma mansoni* infected mice, gut bacterial diversity decreased, and *Akkermansia muciniphila* (phylum *Verrucomicrobia*) and Lactobacillales abundance increased compared to controls [109]. *Trichuris muris* infection in mice decreased the bacterial diversity of the large intestine and an increase the relative abundance of *Lactobacillaceae* was observed. This alteration in the bacterial community structure resulted in greater abundance of *Alistipes*, *Odoribacter*, and *Parasutterella*, and a decrease in *Allobaculum* and *Barnesiella* [35]. A further study using 454 pyrosequencing showed that *Trichuris muris* infection resulted in a decrease in the diversity and abundance of Bacteroidetes, namely *Prevotella* and *Parabacteroides* genera in the faecal bacterial communities in mice [92]. Infection with the trematode *Hymenolepis diminuta* has been associated with a decrease in Actinobacteria and Tenericutes and an increase in Bacteroidetes [110]. In another study, *Hymenolepis diminuta* infection produced a significant change in 48 OTUs, assessed via V4 region 16S metabarcoding, of the gastrointestinal bacterial community of rats [94,95]. The treatment of *H. diminuta* infection triggered an increase in the abundance of uncultured Bacteroidales family S24-7 and Ruminococcaceae and Mollicutes RF39 order. In addition, the genera *Turcibacter* and *Sutterella*, and Erysipelotrichaceae were significantly more abundant in non-infected rats [94]. Illumina MiSeq V4 16S metabarcoding in rats showed that *H. diminuta* infection altered the *Firmicutes* species structure with an increase in *Clostridia* and a decrease in *Bacilli* [95].

Analysis of the duodenal microbiota of rabbits (*Oryctolagus cuniculus*) experimentally infected with *Trichostrongylus retortaeformis* by 454 pyrosequencing V3-V5 16S metabarcoding found an increase in *Leptospiraceae* and *Desulfobacteraceae*, and *Leptomena* and *Desulfocella*, whereas the abundance of *Porphyromonadaceae* and *Bacteroidaceae* was higher in controls [103].

Analysis of the intestinal microbiota in cats experimentally infected with *Toxocara cati* by Illumina MiSeq V3–V4 16S metabarcoding showed that i) Firmicutes, Proteobacteria, Actinobacteria, and Bacteroidetes were abundant in all samples; ii) Gammaproteo-Bacteria, *Jeotgalicoccus*, and *Jeotgalicoccus psychrophilus* were less abundant in infected cats; whereas iii) there was a decrease in *Collinsella stercoris*, *Enterococcus cecorum*, *Ruminococccus gnavus*, *Dorea*, and *Lactobacillales* in non-infected cats [104].

In addition, 16S rRNA metabarcoding showed that *Ascaris suum* infection affects the microbial communities in the faecal and proximal colon of pigs. Significant changes in the abundance of *Prevotella* and *Faecalibacterium* and metabolic pathways have been observed following infection with *Ascaris suum*. A significant positive correlation was found between node connectivity of the operational taxonomic units assigned to *Proteobacteria* (especially the family *Alcaligenaceae*) and faecal acetate and propionate levels. However, the family *Porphyromonadaceae* was positively correlated with faecal egg counts [111]. *Trichuris suis* experimental infection in pigs induced a decreased abundance of *Fibrobacter* and *Ruminococcus* and an increased abundance of *Campylobacter* in the colon microbiota, assessed using Illumina HiSeq 2000 16S metabarcoding [87]. Another study on alterations in the porcine colon microbiota when experimentally infected with *Trichuris suis* showed a decreased abundance of *Ruminococcus*, *Oscillibacter* and *Succinivibrio* and an increased abundance of *Mucispirillum* and *Paraprevotella* using 16S metabarcoding and whole-genome shotgun (WGS) sequencing [88]. The study of the gut bacterial community after therapeutic *Trichuris trichiura* infection of macaques with chronic idiopathic diarrhoea showed an increase the genus *Streptophyta* of the phylum Cyanobacteria using Illumina MiSeq V4 16S metabarcoding [106]. The Illumina MiSeq V3V4, V5V7 16S rRNA metabarcoding of the ovine gut bacterial community at different stages of *Haemonchus contortus* infection showed a relatively increased abundance of *Pseudomonas*, *Ochrobactrum*, *Escherichia*/*Shigella* and *Azotobacter* genera, at the egg stage; followed by *Achromobacter*, *Lentibacillus*, *Pseudomonas*, *Ochrobactrum*, *Kroppenstedtia*, *Dokdonella*, *Bacillus*, *Delftia*, *Oceanobacillus*, *Azotobacter*, *Pseudaminobacter* and *Candidatus Accumulibacter*, at the larval stage; and *Escherichia*-*Shigella*, *Pseudomonas*; and *Ochrobactrum* genera, at the adult stage [98]. V3-V4 16S metabarcoding characterisation of the equine gut commensal flora when infected with low and high numbers of cyathostomin eggs showed that the Methanomicrobia (class) and *Dehalobacterium* (genus) were more abundant in equines experimentally infected with lower egg counts compared to those infected with higher egg counts [100].

(b) Clinical studies

Regarding clinical studies, Illumina MiSeq V4-16S metabarcoding of the gut bacterial community of primary school children from Taiwan showed that *Enterobius vermicularis* infection was associated with increased gut bacterial diversity and mebendazole treatment was associated with a further increased gut bacterial diversity. Enterobiasis and mebendazole deworming were both associated with a relatively high abundance of *Bifidobacterium longum*, *Oscillospira* sp., *Faecalibacterium prausnitzii*, *Alistipes*. Mebendazole treatment induced a relative decrease in *Acidaminococcus intestini*, *Megasphaera elsdenii*, *Veillonella dispar*, *Fusobacterium varium*; and a relative increase in *Collinsella aerofaciens*, and *Streptococcus thermophilus* [34]. In addition, 454 pyrosequencing V3V5 16S metabarcoding of the gut bacterial community in school children in Ecuador found no evidence for changes associated with *Trichiuris trichiura* infection, but mixed *T. trichiura* and *Ascaris lumbricoides* infection was associated with a reduced bacterial diversity and a decreased proportional abundance of a few genera in the *Clostridia* class of *Firmicutes* [89]. Another Illumina MiSeq V4 16S metabarcoding study found higher species richness and abundance of *Paraprevotellaceae*, *Mollicutes*, *Bacteroidales*, and *Alphaproteobacteria* in the gut bacterial community of Malaysian villagers infected with the soil-transmitted helminths, *Trichuris* spp., *Ascaris* spp., and hookworm [90]. In patients with coeliac disease on a gluten free diet, 454 pyrosequencing V1V3-V3V5 16S metabarcoding of the intestinal microbiota showed that experimental *Necator americanus* infection was associated with significant increases in microbial species richness despite maintaining the bacterial composition of the intestinal flora [96]. It has been reported by sequencing of the V3V4 region of the bacterial 16S rRNA with the Illumina MiSeq system that the bacteria of *Verrucomicrobiae*, *Verrucomicrobiales*, *Verrucomicrobiaceae* and *Enterobacteriaceae*, *Lactococcus*, *Akkermansia* and a genus belonging to the *Enterobacteriaceae* and *Akkermansia muciniphila* had increased abundance in patients from Sri Lanka with intestinal helminths (*Ascaris*, *Trichuris*, *Hookworm*). However, *Leuconostocaceae* and *Bacteroidaceae* and *Bacteroides* were less common in patients infected with worms compared to those who were either not infected or under anti-helminthic prophylaxis [36]. A study in Liberia and Indonesia, using Illumina MiSeq and 454 pyrosequencing V1 V3 16S of the gut microbiota metabarcoding, showed that *Lachnospiracae* were associated with the absence of soil-transmitted helminth (*Ascaris lumbricoides*, *Trichuris trichiura*, *Necator americanus*) infection, whereas 12 bacterial taxa were significantly associated with helminth infections, including *Olsenella*, the abundance of which significantly decreased after anthelmintic treatment. Successful anthelmintic treatment was associated with the presence of *Clostridium*_IV, *Turicibacter*, and *Collinsella*. *Akkermansia* and *Ruminococcus* were significantly associated with both infection at baseline and the prevention of parasite clearance [91]. Illumina MiSeq V3V4 16S metabarcoding of the bacterial community in children’s guts showed that *Schistosoma haematobium* infection was associated with decreased abundance of *Firmicutes* and an increased abundance of *Proteobacteria*. In particular, the genus *Prevotella* was significantly more abundant in children infected with schistosomiasis [99]. Another study on children from Ivory Coast who were exposed to *Schistosoma mansoni* used 16S metabarcoding of the bacterial community and revealed that *Schistosoma mansoni* infection in children in Ivory Coast was not associated with gut dysbiosis, but *Fusobacterium* spp. abundance was positively correlated with the clinical efficacy of praziquantel treatment [105].

#### 3.2.3. Interaction between Fungal and Bacterial Communities

##### Impact of Fungi on Bacterial Community Diversity

Like protozoa and helminths, fungi, also in the minority, play a crucial role in the interaction between prokaryotes and eukaryotes in the host.

The presence of the baker’s yeast *Saccharomyces* has been associated with an increase in the diversity of gut bacterial communities in human and animal models [112,113,114,115]. Furthermore, a decrease in *Saccharomyces* spp. abundance has also been correlated with a decrease in bacterial diversity [37,116,117]. Similarly, the ingestion of *Ganoderma lucidum* mycelium in mice with a high-fat diet displayed an anti-obesity effect that was associated with increased bacterial diversity [118]. A non-statistically significant trend towards greater bacterial diversity and a reduction in the richness of fungi has been observed between normal weight and obese children [114]. In patients with chronic diseases, it has been observed that the increase in fungal microbiota diversity has very often resulted in a decrease in bacterial diversity, manifested by a decrease in the abundance of *Bacteroidetes* or *Firmicutes* [38,116,117,119,120,121]. Infection with the microsporidia *Paranosema locustae* was associated with a decrease in bacterial diversity in migratory locusts [122]. Furthermore, *Clostridium difficile* colitis has been associated with a decrease in the diversity of fungal alpha [37]. It was also noted in a study on the characterisation of fungi and bacterial microbiota in Rett Syndrome patients that a high abundance of *Candida* spp. was associated with an increased abundance of the *Clostridia* genus among the bacterial community, when using high-throughput sequencing of 16S rRNA [121]. Furthermore, the existence of great fungal diversity in the Libellulidae *Pantala flavescens*, new symbiotic bacteria *Leclercia* sp., *Oceanobacillus oncorhynchi*, and *Methylobacterium extorquens* has been described. The existence of an antibacterial activity of symbiotic fungi in the *Pantala flavescens* larvae has also been demonstrated [123]. It is notable that a decrease in *Malassezia* abundance and a decrease in bacterial diversity have been seen in children with Hirschsprung disease and in Rett Syndrome patients [117,121]. However, an increased abundance of *Malassezia sympodialis* associated with a decrease in bacterial diversity has been observed in children with inflammatory bowel disease [116]. In the current literature, fungi are reported to influence the bacterial communities and tend towards a relatively healthy state. For instance, *Saccharomyces* spp. improves gastrointestinal discomfort and constipation, *Ganoderma lucidum* mycelium displays anti-obesity properties, and symbiotic fungi have antibacterial activity. All these fungal properties have been associated with the modulation of the diversity and structure of the host bacterial community [112,114,118,123,124].

##### Impact of Fungi on Bacterial Community Structure

Fungi are relatively numerous and ubiquitously colonize the living environment where they modulate the bacterial microbiota (Table 3). Hence, a growing body of evidence indicates that fungi may modulate the microbiota and play important roles in the physiology and immunity of the host [116]. In the literature, studies of the interaction between fungus and bacteria mainly concern yeasts, including *Candida albicans* and the probiotics *S. cerevisiae* RC016 and *Saccharomyces boulardii*, filamentous fungi including *Mucor circinelloides*, macroscopic fungi, such as *Ganoderma lucidum*, and microsporidian enteropathogens.

(a) In vitro studies

The in vitro study of *Candida albicans* and *Clostridium difficile* interactions showed that the strictly anaerobic Gram-positive bacterium *C. difficile* can grow under aerobic conditions in the presence of *C. albicans*. In contrast, *C. albicans* hyphal formation is inhibited by the presence of *C. difficile*, most probably due to p-cresol excretion [124]. Another study on anaerobic bacteria/yeast interactions highlighted that the growth of *Bacteroides* was significantly enhanced in co-culture with *C. albicans*, whereas the growth of *C. albicans* was affected neither by *B. fragilis* nor *B. vulgatus* co-culture, suggesting that *C. albicans* cells can serve as an additional nutrient source for the culture of bacteria in the anaerobic atmosphere of the gut [126].

(b) Experimental studies

An in vivo study, using a mouse model of obesity, showed through V3-V5 16S rRNA metabarcoding that 4% to 8% water extract of the basidiomycete *Ganoderma lucidum* (WEGL) mycelium reduces the *Firmicutes*-*Bacteroidetes* and *Proteobacteria* ratio in high-fat diet mice. Treatment of high-fat diet-fed mice with 8% WEGL increased the abundance of the species *Parabacteroides goldsteinii*, *Bacteroides* spp., *Anaerotruncus colihominis*, *Roseburia hominis*, *Clostridium* spp., *Methylpentosum* (*Clostridium* IV), *Clostridium* XIVa and XVIII, and *Eubacterium coprostanoligenes*, which were negatively correlated to obesity. In addition, 8% WEGL increased the abundance of the species *E. coprostanoligenes*, *C. methylpentosum*, *P. goldsteinii*, *Bacteroides* spp., *A. colihominis*, *R. hominis*, and *Clostridium* XIVa and XVIII in Chow diet mice [118]. Other in vivo studies using mouse models have analysed the interaction between yeasts and the gut microbiota. Feeding with the probiotic *S. cerevisiae* RC016 was associated with a decrease of one logarithmic unit of *Enterobacteriaceae* in healthy mice compared with control mice using conventional culture methods [134]. Moreover, some studies have revealed cooperation between the *Enterobacteriaceae* family and both *C. albicans* and *S. boulardii*, encouraging their gut colonisation and their effect on intestinal inflammation. The colistin-resistant *Escherichia coli* effect revealed the beneficial impact of *S. boulardii* and pathogenic effects of *C. albicans* on colitis severity in mice [125]. Faecal microbiota transplantation can prevent fungal colonization of the gastrointestinal tract. In fact, the experimental wild-type mice model is resistant to gut colonization by *Candida albicans*. Mice treated with β-lactam antibiotics (e.g., Ampicillin) experienced a dysbiosis in the gut microbiota which was beneficial to *Candida albicans* by colonising the digestive tract. Faecal microbiota transplantation effectively and immediately reduces *C. albicans* loads and prevents it from colonising the gastrointestinal tract of mice [136]. Specific probiotics such as *Bifidobacterium* may also be of therapeutic interest by reducing the fungal load in *Candida albicans*/*Clostridum difficile* infection models. In fact, the administration of *Candida albicans* aggravates the severity of *Clostridium difficile* infection by increasing gut inflammation. Unfortunately, the probiotic has no effect on the clostridium toxin in the faeces [128]. Colonisation by *C. albicans* does not always have a deleterious effect, showing protective effects against lethal *C. difficile* infections in mice models by acting on the cytokine IL-17A. The abundance of the beneficial bacteria *Bifidobacterium* and *Akkermansia* was significantly increased in mice colonised with *C. albicans* [127]. Another pathogenic opportunistic yeast, *Candida glabrata*, in an experiment using a colitis mouse model showed that the persistence of *C. glabrata* in the gut is subject to remodelling its cell wall leading to an increase in chitin. Oral administration of chitin restored anaerobic bacteria including *Lactobacillus reuteri*, *Lactobacillus johnsonii*, *Bifidobacterium* and *Bacteroides* spp. and reduced aerobic bacteria such as *Escherichia coli* and *Enterococcus faecalis*, countering the effect of intestinal inflammation caused by colitis [137]. The treatment of rats with *Saccharomyces cerevisiae* fermentation prebiotic before stress leads to beneficial changes in the gut microbiota. Treating rats with yeast fermentate before exposure to heat stress resulted in changes in the relative abundance of *Bifidobacterium* and *Allobaculum*, while *Acetanaerobacterium*, *Bacteroides*, *Eubacterium*, *Johnsonella*, *Lactococcus*, *Oscillospira*, *Roseburia* and *Vallitalea* substantially increased. [135]. The usefulness of *Saccharomyces boulardii* CNCM I-745 probiotics has been studied in other pathologies, through a controlled study of the lipid profile and the intestinal microbiota in a hypercholesterolemic hamster model. The abundance of the genus *Allobaculum* increased and an unclassified genus in the family *Lachnospiraceae*, unclassified genus of *Desulfovibronaceae*, *Oxalobacter* and an unclassified genus in family F16 with the treatment of *Saccharomyces boulardii* CNCM I- 745 decreased with treatment of *Saccharomyces boulardii* CNCM I-745. These genera g_CF231, *Allobaculum*, an unclassified *Lachnospiraceae* and *Oxalobacter* have been correlated with total plasma cholesterol [133]. Elsewhere, a study investigated the effect of (3R, 30R)-astaxanthin on lipid metabolism and the gut microbiota in mice fed on a high-fat diet. Astaxanthin is produced from *Xanthophyllomyces dendrorhous*, a basidiomycete fungus. Supplementation with (3R, 3′R)-astaxanthin/*X. dendrorhous* on a high-fat diet prevented weight gain and decreased total cholesterol in the plasma and liver. Furthermore, it regulated its intestinal microbiota optimising the *Bacteroidetes*/*Firmicutes* ratio and increasing the abundance in *Verrucomicrobia*, particularly *Akkermansia* [138]. It has also been shown in a mouse model treated with the pathogenic fungus *Mucor circinelloides* that the abundance of the bacterial genus *Bacteroides* increases, and the abundance of the bacteria *Akkermansia muciniphila* decreases in these gastrointestinal tracts [131]. The administration of mushrooms (*Agaricus bisporus*) to pigs significantly reduced the *Salmonella typhymurium*-Lipopolysaccharide-induced inflammatory response at the alveolar macrophage level and positively modulated the metabolism of the pig microbiota by increasing the abundance of *Clostridial* taxa which are associated with improved intestinal health [130].

In vivo studies have also been carried out using insect models. The microbiota of healthy dragonfly (*Pantala flavescens*) larvae was analysed using culture-dependent methods and ITS barcoding for the fungi, and 16S barcoding for the bacterial symbionts; forty-eight fungal isolates were obtained, grouped in five classes (Leotiomycetes, Dothideomycetes, Eurotiomycetes, Sordiaromycetes, Zygomycetes), were associated with a variety of symbiont bacteria, including *Sphingomonas*, *Methylobacterium*, *Burkholderia*, *Pantoea*, *Enterobacter*, *Leclercia*, and *Serratia*, *Oceanobacillus* which were included in the *Proteobacteria* and *Firmicutes*. *Enterobacter* was the most abundant bacterial genus associated with these fungi [123]. In honey bees (*Apis mellifera*), *Nosema ceranae* microsporidium infection was associated with a decreased abundance of *Alphaproteobacteria*, *Bifidobacterium* spp. and *Lactobacillus* spp., an increased abundance of *Gilliamella apicola*, and *Snodgrassella alvi* increased significantly in honeybees infected during the winter assessed using quantitative real-time PCR (qPCR) [132]. In vivo insect models have also been used to study the impact of microsporidian parasites on the gut microbiota. Thus, in locusts (*Locusta migratoria*), infection with the microsporidian parasites *Paranosema locustae* alters the structure of the gut bacterial community, as assessed using 16S rRNA V4–V5 region pyrosequencing, by increasing the abundance of the genera *Citrobacter* (36%), *Lactococcus* (13.28%) and *Raoultella* (43%) [122]. In *Serinus canaria* birds, colonisation of the gastric mucosa by the opportunistic yeast *Macrorhabdus ornithogaster* was associated with the presence of *Lactobacillus* and *Candidatus Arthromitus*, assessed via 16S metabarcoding, whereas *Lactococcus*, *Pseudomonas*, *Acinetobacter*, *Lachnospiraceae*, *Propionibacterium* and *Weissella* were associated with uninfected birds [129].

(c) Clinical studies

The data on interactions between fungal and bacterial communities in humans remain scarce. Analysis of the diversity of bacterial and fungal communities in normal-weight and obese school-aged children showed the presence of *Eubacterium rectale*, *Saccharomyces cerevisiae*, *Candida albicans*, and *C. glabrata* in all subjects, whereas there was a significantly lower abundance of *Akkermansia muciniphila*, *Faecalibacterium prausnitzii*, *Bacteroides*/*Prevotella* Group, *Candida* spp., and *Saccharomyces* spp. in obese children, and *Debaryomyces hansenii* was found in two obese children [114]. Another study using both 16S and ITS rRNA metabarcoding analysed the impact of the antibiotic treatment of *Clostridium difficile* infection (CDI) on bacterial and fungal communities. It showed a relatively increased abundance of the *Pichiaceae* family (order of Saccharomycetales) in non-CDI patients and, in contrast, a relatively increased abundance of the *Ascomycota* phylum, the *Pleosporales* order, and the *Dothideomycetes* class in the patients with CDI. A relatively increased abundance of *Ascomycota* phylum and *Saccharomycetes* was observed in patients with CDI. The genera *Cadophora*, *Bandoniozyma* and *Clitocybe* were more abundant in the non-CDI than in CDI patients [37]. Another study looked at the effect of oral administration of *Saccharomyces boulardii* and its mode of administration on the intestinal microbial community in premature infants by 16S metabarcoding. The results showed that *Firmicutes* and *Proteobacteria* remained stable during the observation period. The oral administration of *Saccharomyces boulardii* had no significant influence on the bacterial community but the mode by which children were delivered changed the microbiota. *Bacteroides* and *Parabacteroides* were more abundant in children delivered vaginally compared to children born by Caesarean delivery on day 0. After two weeks of administration, the abundance of *Bacteroides* and *Parabacteroides* were higher in children delivered vaginally compared to children born by Caesarean section [115]. In women treated for bacterial vaginosis, the effect of the antibiotic treatment was reduced by *Saccharomyces boulardii* prophylaxis, thus improving the colonic microbiota [139]. Elsewhere, in patients with inflammatory bowel disease, a positive correlation was observed between the abundance of *Saccharomyces* and that of *Bifidobacterium*, *Blautia*, *Roseburia* and *Ruminococcus* using both ITS2 and 16S rRNA metabarcoding [116]. Some studies have focussed on bacterial and fungal microbiota in patients with chronic inflammatory bowel disease. Patients with primary sclerosing cholangitis suffer from fungal microbiota dysbiosis, an alteration in composition and high biodiversity. An increased proportion of *Exophiala* and a decreased proportion of *Saccharomyces cerevisiae* has been observed among these patients. The signature of fungi dysbiosis is different when compared with patients with Irritable Bowel Disease. The bacteria–fungi correlation network highly affects the intestinal microbiota of patients with primary sclerosing cholangitis when compared to patients with Irritable Bowel Disease status. Gut fungi could therefore contribute to the pathogenesis of primary sclerosing cholangitis and could be considered as a new therapeutic target [140]. Some species such as *Candida tropicalis*, *Serratia marcescens* and *Escherichia coli* have been found to be associated with Crohn’s disease dysbiosis [120]. Meanwhile, another study found that the genera *Candida*, *Debaryomyces*, *Saccharomyces*, *Malassezia*, *Sporobolomyces*, *Trichosporon*, *Wallemia*, unidentified *Filobasidiaceae* and unidentified *Xylariale* as well as the genus *Enterococcus*, *Alicyclobacillus* and *Lactobacillus* were over-represented in patients with Crohn’s disease using 16S rRNA (MiSeq) and ITS2 (pyrosequencing) [38]. The relative abundance of *Bifidobacterium* and several *Clostridia* including *Anaerostipes*, *Clostridium* XIVa, and *Clostridium* XIVb, as well as *Erysipelotrichaceae Clostridium* XVIII and *Erysipelotrichaceae incertae sedis*), *Actinomyces*, *Eggerthella*, *Enterococcus*, *Escherichia*/*Shigella* and *Lactobacillus* were higher in the Rett Syndrome patients compared with healthy controls when using high-throughput sequencing the V3-V5 regions of the 16S rDNA gene. The gut fungal community, analysed by sequencing the ITS1 region of the rRNA, revealed the most abundant genera in Rett Syndrome patients were *Candida*, *Aspergillus* and *Trichosporon*, whereas in healthy controls *Penicillium*, *Malassezia*, *Debaryomyces*, *Mucor*, *Eremothecium*, *Pichia* and *Cyberlindnera* were the most abundant. The genus *Candida* was significantly more abundant in Rett Syndrome patients than in healthy controls [121]. It has been reported, using MiSeq Illumina ITS-1 sequencing, that *Candida* species in the Hirschsprung disease group was composed of *C. albicans*, *C. tropicalis*, *C. parapsilosis* and *C. utilis*, while the Hirschsprung-associated enterocolitis group had a majority of *C. albicans* and low *C. tropicalis*. Ion Torrent 16S rRNA sequencing revealed a low proportion of *Firmicutes* and *Verrucomicrobia* and a higher proportion of *Bacteroidetes* and *Proteobacteria* in the Hirschsprung-associated enterocolitis group, when compared to the Hirschsprung disease group [117]. Analysis of the fungal microbiota by the shutgun metagenomic of a cohort of colorectal cancer (CRC) patients, adenoma patients and control subjects from Hong Kong. Results showed that CRC was associated with fungal microbiota dysbiosis with an increased Basidiomycota:Ascomycota ratio in CRC patients compared to healthy subjects. It was also reported that Malasseziomycetes were increased in CRC while Saccharomycetes and Pneumocystidomycetes were decreased. Nevertheless, CRC patients that are enriched in Geobacteraceae, Synergistaceae, Peptoniphilacea and Fusobacteriaceae were found to have a positive correlation between CRC enriched in fungi Chaetomiaceae and CRC decreased in Ruminicoccaceae; a negative correlation between CRC enriched in fungi Pseudeurotiaceae and CRC enriched in Geobacteriaceae. These results suggested that altered fungal composition may play a role in CRC [141]. Food consumption has been associated with fungal abundance in the gut. An inverse association between *Candida* (fungus) and *Bacteroides* (bacteria) has been found. A higher abundance of *Bacteroides* has been observed in individuals whose diet is very high in protein, while *Candida* is more highly abundant in individuals who have recently consumed carbohydrates. The authors have also reported a positive correlation between *Fusarium* (fungus), *Bryantella* (bacteria) and *Anaerostipes* (bacteria), and *Pichia* (fungus) and *Syntrophococcus* (bacteria) [142,143].

## 4. Conclusions and Perspectives

While the “One Health” concept acknowledges that human health is linked to animal health and to the environment [144,145], microbial community structures are dependent on interactions between each of their components. Studies addressing only one component, for instance the bacterial community, are limited by only providing a partial view of both the structure of micro-organism communities and the inter-kingdom interactions between communities of sympatric viruses, prokaryotes and eukaryotes. The human and animal microbiota biotope include the bacterial, viral, eukaryotic (protozoa and helminths) and fungal communities. These communities of micro-organisms coevolve and maintain balanced relationships in the host. The relationship between prokaryotic and eukaryotic communities and their environment contributes to homeostasis and host health. This relationship is altered by the qualitative and quantitative modification of microbiota that are, among other factors, influenced by anti-infective treatments, genetic predisposition, and digestive and chronic diseases. Studies analysing the interaction between communities of prokaryotes and eukaryotes are scarce. However, large-scale controlled studies are needed to elucidate the mechanisms that explain variations in the diversity and abundance of the prokaryotic microbiota resulting from the presence of eukaryotes.

This review highlights that some bacteria, especially *Lactobacillus* sp., have been associated with the inhibition of infection by the protozoa *Giardia duodenalis* in vitro and *Plasmodium falciparum* in vivo. Infection of the intestinal protozoa has a qualitative and quantitative impact on the intestinal microbiota. Free-living amoebae maintain symbiotic relationships with most microorganisms such as virus, bacteria, fungi and parasites. Several worms have been involved in alterations in bacterial communities. Infecting wild-type C57BL/6 mice with *Heligmosomoides polygyrus bakeri* significantly increased the abundance of the *Lactobacillaceae* family, but the clinical consequences of these changes in the intestinal flora have yet to be studied. Fungi can be used as a probiotic (EpiCor fermentate) to modulate the microbiota especially the bacterial community by improving gastrointestinal discomfort and constipation. Some studies have demonstrated that fungi are associated with modulation of the microbiota in chronic diseases as well as in cases of HIV and CDI infections.

The impact on host immunity and the metabolic potential of changes to the microbiota has not been addressed in this review.

The interaction between eukaryotes and prokaryotes resulted in modulation of the microbiota which led to the characterisation of the complexity of the microbiome. This interaction could play a significant role in the pathophysiology of various multifactorial chronic diseases. It is thus important to further study the structure and function of both prokaryotic and eukaryotic communities to better understand their interactions. The microbiota community structure has been characterised by the development of metagenomic/genomic and related culture methods. However, further research is warranted to bridge the knowledge gap on interactions between eukaryotes and prokaryotes.

## Figures and Tables

**Table 1:** Impact of protozoa on bacterial community structure. The meaning of ↑ is increased and ↓ decreased.

Protozoa	Host	Type of Sample	Site of Sample	Bacterial Microbiota Method		Bacterial Microbiota Change	Diversity Profile	Reference
*Blastocystis* spp.	Cirrhotic patients	Faeces		High-throughput sequencing of 16S rRNA amplicons (Illumina)		Family: Enterobacteriaceae↑, Ruminococaceae↓ Genus: *Lactobacillus*↑, clostridial cluster XIV↓	↓alpha diversity	[74]
	Healthy human			High-throughput sequencing of 16S rRNA amplicons (Ion Torrent)		Class: Clostridia↑, Mollicutes↑ Order: Clostridiales↑, Lactobacillales↓ Family: Enterobacteriaceae↓, Enterococcaceae↓, Streptococcaceae↓, Lactobacillaceae↓, Ruminococcaceae↑, Prevotellaceae↑ Genus: *Acetanaerobacterium*↑, *Acetivibrio*↑, *Coprococcus*↑, *Hespellia*↑, *Oscillibacter*↑, *Papillibacter*↑, *Sporobacter*↑, *Ruminococcus*↑, *Prevotella*↑, *Roseburia*↓, *Faecalibacterium*↓	↑alpha diversity	[29]
				High-throughput sequencing of 16S rRNA amplicons (Illumina)		Phylum: Firmicutes↑, Elusimicrobia↑, Lentisphaerae↑, Euryarchaeota↑, Actinobacteria↓, Proteobacteria↓, unassigned bacteria↓, Deinococcus–Thermus↓ Class: Clostridia↑, IHU_PC_PC_Bacteria↑, Elusimicrobia↑, Lentisphaeria↑, Metanobacteria↑, Deltaproteobacteria↑, Planctomycetacia↓, Rubrobacteria↓, Deinococci↓, Gammaproteobacteria↓, Actinobacteria↓, unassigned bacteria↓, Bacilli↓ Order: Clostridiales↑, IHU_PO_Bacteria↑, Victivallales↑, Methanobacteriales↑, Elusimicrobiales↑, Aeromonadales↑, Acidaminococcales↑, Desulfovibrionales↑, Planctomycetales↓, Rhodobacterales↓, Sphingomonadales↓, Rubrobacterales↓, Veillonellales↓, Pasteurellales↓, Micrococcales↓, Pseudonocardiales↓, Enterobacteriales↓, Myxococcales↓, Bifidobacteriales↓, unassigned bacteria↓, Lactobacillales↓ Family: Clostridiaceae↑, Ruminococcaceae↑, Lachnospiraceae↑, Streptococcaceae↓, Bifidobacteriaceae↓, Enterobacteriaceae↓, Leuconostocaceae↓ Genus: *Ruminococcus*↑, *Clostridium*↑, *Streptococcus*↓, *Bifidobacterium*↓, *Shigella*↓ Species: *Clostridium saudii*↑, *Methanobrevibacter smithii*↑, *Streptococcus* sp.↓, *Bifidobacterium* sp.↓, *Shigella* sp.↓	↑alpha diversity	[80]
*Blastocystis* spp. with or not *Dientamoeba fragilis*	Healthy human	Faeces		qPCR		Genus: *Bacteroides*↓, Clostridial cluster XIVa↓, *Prevotella*↑	Not evaluated	[27]
*Cryptosporidium parvum*	CD-1 mice	Faeces		High-throughput sequencing of 16S rRNA amplicons (Illumina)		Phylum: Unclassified Bacteroidetes↑ Family: Porphyromonadaceae↑, Prevotellaceae↑	Not Change	[73]
						Phylum: Proteobacteria↑, Firmicutes↓	↓alpha diversity	[81]
*Entamoeba histolytica*	Children	Faeces		Quantitative polymerase chain reaction (qPCR)		Species: *Prevotella copri*↑	Not evaluated	[31]
*Entamoeba* (*E. dispar*, *E. histolytica*, or both)	Pygmy hunter-gatherers Bantu individuals			High-throughput Sequencing of 16S rRNA amplicons (Illumina)		Phylum: Actinobacteria↓, Bacteroidetes↓, Cyanobacteria↑, Elusimicrobia↑, Euryarchaeota↑, Firmicutes↑, Fusobacteria↓, Lentisphaerae↓, Spirochaetes↑, Tenericutes↑, Verrucomicrobia↑ Order: Clostridiales↑, Elusimicrobiales↑, Treponema↑ Family: Christensenellaceae↑, Elusimicrobiaceae↑, Spirochaetaceae↑ Specie: *Prevotella copri*↓	↑alpha diversity	[75]
*Giardia* *duodenalis*, *Ancylostoma* *caninum*, *Cystoisospora*, *Giardia cati*	Dog Cat	Faeces		High-throughput sequencing of 16S rRNA amplicons (Ion Torrent)		Dog: *Giardia* Phylum: Firmicutes↑, Bacteroidetes↓, Proteobacteria↑ Family: Erysipelotrichaceae↑, Bacteroidaceae↓, Lachnospiraceae↓, Pseudomonadaceae↑ Genus: *Catenibacterium*↑, *Pseudomonas*↑, *Howardella*↑, *Bacteroides*↓, *Pseudobutyrivibrio*↓,	No change	[79]
						Cat: *Cystoisospora* Phylum: Actinobacteria↑, Firmicutes↑ Deinococcus-Thermus↑, Proteobacteria↑ Family: Bifidobacteriaceae↑, Coriobacteriaceae↑, Veillonellaceae↑, Bacillaceae↑, Thermaceae↑, Xanthomonadaceae↑, Comamonadaceae↑, Beijerinckiaceae↑, Xanthomonadaceae↑ Genus: *Bifidobacterium*↑, *Olsenella*↑, *Megamonas*↑, *Geobacillus*↑, *Meiothermus*↑, *Bacillus*↑, *Thermomonas*↑ *Schlegelella*↑, *Chelatococcus*↑, *Silanimonas*↑		
						Cat: *Giardia* Phylum: Firmicutes↑ Family: Lachnospiraceae↑, Ruminococcaceae↓ Genus: *Roseburia*↑, *Subdoligranulum*↓		
*Giardia lamblia*	C57BL/6J mice	Mucosal and Luminal proximal Small intestine, Mucosal and luminal Distal small intestine, Cecal contents and colonic contents	Foregut, hindgut	High-throughput sequencing of 16S rRNA amplicons (Illumina)		Phylum: Melainabacteria↓ Order: Clostridiales↓, Family: Rhodocylaceae↑, Moraxellaceae↑	↓alpha diversity	[82]
*Giardia duodenalis*	Healthy human	Mucosal biopsies	Colon	High-throughput sequencing of 16S rRNA amplicons (Illumina)		Phylum: Firmicutes↑ Order: Clostridiales↑ Genus: *Phascolarctobacterium*↓	↑alpha diversity	[30]
*Giardia* spp., *Entamoeba* spp./ *Blastocystis hominis*	Human with or without symptoms	Faeces		qPCR		Genus: *Bifidobacterium*↑, Species: *Escherichia coli*↑, *Faecalibacterium prausnitzii*-/*Escherichia coli* ratio↑	Not evaluated	[28]
*Leishmania infantum*	Lutzomyia longipalpis		midguts	16Sv4 rRNA gene sequencing		Family: Pseudomonadaceae↑, Acetobacteraceae↓	↓alpha diversity	[76]
*Plasmodium yoelii*	BALB/c mice Resistant, C57BL/6 mice susceptible	Cecum, colon	Distal small intestine	High-throughput sequencing of 16S rRNA amplicons (Illumina)		Phylum: Firmicutes↑, Bacteroidetes↓ Family: Clostridiaceae↑, Erysipelotrichaceae↑, Lactobacillaceae↑, Peptostreptococcaceae↑ Bacteroidaceae↓, Prevotellaceae↓, Sutterellaceae↓ Genus: *Lactobacillus*↑, *Bifidobacterium*↑	↓alpha diversity	[33]
*Trichomonas vaginalis*	North American women	Vaginal swabs		High-throughput Sequencing of 16S rRNA amplicons (454 pyrosequencinq)		Genus: *Lactobacillus*↓, *Mycoplasma*↑, *Parvimonas*↑, *Sneathia*↑	Not evaluated	[78]
*Toxoplasma gondii*	NOD2^−/−^ mice	Feces			Real Time-PCR	Order: Enterobacteria↑ Genus: *Lactobacillus*↑, *Bifidobacteria*↓, *Enterococci*↑, *Bacteroides*/*Prevotella* spp.↑, eubacterial↑	Not evaluated	[77]
	C57BL/6 mice				High-throughput sequencing of 16S rRNA amplicons (Illumina)	Phylum: Firmicutes↑ Genus: *Clostridia*↑		[83]

**Table 2:** Influence of helminths on bacterial community structure. Note that ↑ is increased and ↓ decreased.

Helminths	Host	Type of Sample	Site of Sample	Bacterial Microbiota Method	Bacterial Microbiota Change	Diversity Profile	Reference
*Ascaris*, *Trichuris*, Hookworm	Human volunteers	Fresh stool		High-throughput sequencing of 16S rRNA amplicons (Illumina)	Class: Verrucomicrobiae↑ Order: Verrucomicrobiales↑ Family: Bacteroidaceae↑, Prevotellaceae↓ Verrucomicrobiaceae↑, Enterobacteriaceae↑, Leuconostocaceae↓, Bacteroidaceae↓ Genus: *Lactococcus*↑, *Akkermansia*↑, *Enterobacteriaceae*↑, *Bacteroides*↓ Species: *Prevotella copri*↑	No change	[36]
*Ascaris* *lumbricoides* (“*Ascaris*”), *Necator* *Americanus* (“*Necator*”), *Trichuris trichiura* (“*Trichuris*”)	Subject cohort	Feces		High-throughput sequencing of 16S rRNA amplicons (Illumina)	Phylum: Firmicutes↑, Bacteroidetes↓, Actinobacteria↑ Family: Lachnospiraceae↑, Erysipelotrichaceae↑ Genus: *Succinivibrio*↑, *Solobacterium*↑, *Desulfovibrio*↑, *Allobaculum*↑, *Rhodococcus*↑, *Lachnospiraceae incertae sedis*↑, *Olsenella*↑, *Flavonifractor*↑, *Enterococcus*↑.	↑alpha diversity	[91]
*Ascaris suum*	Pigs	Digesta	Proximal Colon	High-throughput Sequencing of 16S rRNA amplicons (Illumina)	Genus: ↑*Prevotella*, ↑*Facklamia*, ↑, *Turicibacter*, ↓*Ruminicoccus*, ↓*Lactobacillus*, ↑*Treponema*, ↑*Campylobacter*	↑alpha Diversity with Basal diet No change with Grape pomace diet	[93]
*Cyathostomins* spp. (Eggs hight versus low)	Equines	Feces		High-throughput Sequencing of 16S rRNA amplicons (Illumina)	Class: Methanomicrobia↓, Dehobacterium↓	No difference	[100]
*Enterobius* *vermicularis*	School children	Feces		High-throughput Sequencing of 16S rRNA amplicons (Illumina)	Phylum: ↓Fusobacteria, ↑Actinobacteria Genus: ↑*Bifidobacterium*, ↑*Alistipes*, ↑*Faecalibacterium*, ↓*Fusobacterium*, ↓*Veilonella*, ↓*Megasphaera*, ↓*Acidaminococcus* Species: ↑*Faecalibacterium prausnitzii*, ↑*Ruminococcus flavefaciens*, ↑*Alistipes purtredinis*, ↑*Bifidobacterium longum*, ↑uncultured *Oscillospira* sp., ↓*Acidaminococcus intestine*, ↓*Megasphaera elsdenii*, ↓*Veillonella dispar*, ↓*Fusobacterium varium*	↑alpha diversity	[34]
*Heligmosomoides* *Polygyrus* *bakeri*	IL-4Rα^−/−^ mice; C57BL/6 mice	Lumen	Caecum; ileum; colon	Culture; Cloned 16S rRNA amplicon; qPCR; Denaturing gradient gel electrophoresis	γ-Proteobacteria/Enterobacteriaceae ratio↑, *Bacteroides*/*Prevotella* ratio↑	Not evaluated	[101]
*Heligmosomoides* *Polygyrus*, *Syphacia* spp., *Hymenolepis* spp.	Wild mouse (*Apodemus* *flavicollis*)	Lumen, mucosa	Stomach, ileum, caecum, colon	High-throughput Sequencing of 16S rRNA amplicons (454)	*Hymenolepis* spp. Family: Ruminococcaceae↓, Acetobacteraceae↓, Sphingomonadaceae↓, S24-7 family (Bacteroidetes)↑	No change	[102]
					*Heligmosomoides Polygyrus* Family: Lachnospiraceae↑, S24-7 OTUs (Bacteroidetes)↑ Genus: *Lactobacillus*↑		
					*Syphacia* spp. Family: Lachnospiraceae↓, Lactobacillaceae↓, S24-7 family (Bacteroidetes)↓ Genus: *Lactobacillus*↓		
*Leidynema* *appendiculatum*; *Leidynema* *appendiculatum*; *Hammerschmidtiella* *diesingi*; *Thelastoma* *bulhoesi*	*Periplaneta* *fuliginosa* *Periplaneta* *americana*	Faeces	Foregut; Midgut; Hindgut	High-throughput Sequencing of 16S rRNA amplicons (Illumina)	Phylum: ↓Firmicutes, ↑Proteobacteria, ↓Bacteroidetes, ↑Actinobacteria Genus: ↑*Bacillales*, ↑*Brevibacterium*, ↓*Gordonia*, ↑*Xylanimicrobium*, ↓*Bacteroides* Order: ↑Lactobacillales, ↓Enterobacteriales Family: ↓Lachnospiraceae, ↓Ruminococcaceae, ↑Porphyromonadaceae, ↑Desulfovibrionaceae, ↓Weeksellaceae, ↓Bacteroidaceae *Periplaneta americana* Phylum: ↑Bacteroidetes, ↑Firmicutes, ↑Proteobacteria Family: ↑Porphyromonadaceae, ↓Bacteroidaceae, ↑Ruminococcaceae, ↓Lachnospiraceae, ↑Desulfovibrionaceae	↑alpha diversity	[97]
*Necator* *americanus*	Patients with coeliac disease	Faeces		High-throughput Sequencing of 16S rRNA Amplicons (454)	Phylum: Firmicutes↓, Bacteroides, ↑Tenericutes, RF39↓ Class: Bacteroidia↑, Erysipelotrichi↓, Clostridia↓ Genre: *Ruminococcus*↓, *Lachnospira*↓	↑alpha diversity	[96]
Trichuris muris	C57BL/6 mice	Faeces, lumen	Caecum	High-throughput Sequencing of 16S rRNA amplicons (Illumina)	*Alistipes*, *Odoribacter*, and *Parasutterella*↑, *Allobaculum*↓, *Barnesiella*↓	↓alpha diversity	[35]
		Faeces		Denaturing gradient gel electrophoresis; High-throughput sequencing of 16S rRNA amplicons (454)	Phylum: *Bacteroidetes*↑; Genus: *Prevotella*↑, *Parabacteroides*↑		[92]
*Trichostrongylus* *retortaeformis*	Rabbits (*Oryctolagus* *cuniculus*)	Mucosa	Duodenal	High-throughput Sequencing of 16S rRNA amplicons (Illumina)	Phylum: ↑Proteobacteria, ↑Spirochaetes, ↓Firmicutes Family: Leptospiraceae↑, ↑Ruminococcaceae, ↑Phyromonadaceae, ↑Desulfobacteraceae, ↑Bacteroidaceae Genus: ↑*Leptomena*, ↑*Desulfocella*, ↓*Bacteroides* ↓*Ruminococcus*	↓alpha diversity	[103]
*Toxocara cati*	Cat (*Felis catus*)	Faeces		High-throughput Sequencing of 16S rRNA amplicons (Illumina)	Phylum: ↑Actinobacteria Class: ↑Coreobacteriia, ↓Gammaproteobacteria Order: ↑Lactobacillales, ↑Coribacteriales Family: ↑Enterococcaceae, ↑Coreobacteriaceae Genus: ↑*Collinsella*, ↑*Enterococcus*, ↑*Dorea*, ↑*Lactobacillus*, ↑*Ruminococcus*, ↓*Bulleidia*, ↓*Jeotgalicoccus*	No change	[104]
*Trichuris suis*	Pigs (*Sus* *scrofa* *domestica*)	Faeces	Proximal colon	Whole metagenome shotgun Sequencing (Illumina)	Phylum: ↓Fibrobacteres, ↓Spirochaetes, ↓Tenericutes, ↓ Gemmatimonadetes Genus: ↑*Fibrobacter*, ↑*Campylobacter*, ↓*Treponema*, ↓*Dorea*, ↓*Ruminococcus*	Not evaluated	[87]
	Pigs (*Sus* *scrofa* *domestica*)	Lumen	Proximal colon	Whole metagenome shotgun 454 sequencing High-throughput sequencing of 16S rRNA amplicons (454)	Phylum: ↓Proteobacteria, ↓Deferribacteres, ↑Euryarchaeota Genus: ↑*Prevotella*, ↓*Succinivibrio*, ↓*Mucispirillum*, ↓*Oscillibacter*, ↑*Paraprevotella*, ↑Desulfovibrio, ↑Heliobacter	No change	[88]
*Trichuris* *trichiura*, *Ascaris* *lumbricoides*	School children	Faeces		High-throughput Sequencing of 16S rRNA Amplicons (454)	Phylum: ↓Firmicutes Class: ↓Clostridia, ↑streptococci Genus: ↓*Clostridium sensu stricto*, ↓uncharacterised clostridial cluster IX, ↑*Streptococcus*, ↓*Roseburia*	↓alpha diversity	[89]
*Trichuris* spp., *Ascaris* spp., hookworm	Indigenous community	Faeces		High-throughput Sequencing of 16S rRNA Amplicons (Illumina)	Phylum: ↑Mollicutes, ↑Bacteroidales, ↑Alphaproteobacteria Family: ↑Paraprevotellaceae, ↑Lachnospiraceae, ↑Prevotellaceae Genus: ↓*Bifidobacterium*	↑alpha diversity	[90]
*Schistosoma* *haematobium*, *Schistosoma* *mansoni*	Children (six months to 13 years old)	Urine, Stool		High-throughput sequencing of 16S rRNA amplicons (Illumina)	Phylum: Bacteroidetes↑, Firmicutes↑, Proteobacteria↑ Genus: *Prevotella*↑	↑alpha diversity	[99]
*Schistosoma mansoni*	Children from Côte d’Ivoire	Faeces		High-throughput sequencing of 16S rRNA amplicons (Illumina)	Phylum: Proteobacteria↑ Family: Cerasicoccaceae↑, Anaeroplasmataceae↑, Campylobacteraceae↑, Peptococcaceae↑ Genus: *Klebsiella*↑, *Enterobacter arachidis*↑, *Fructobacillus*↓	↓alpha diversity	[105]
*Trichuris trichiura*	Rhesus monkeys		Colon mucosa	High-throughput sequencing of 16S rRNA amplicons (Illumina)	Phylum: ↓Cyanobacteria, ↑Firmicutes, ↑Bacteroidetes, ↑Tenericutes, ↓unclassified bacteria taxon ZB2 Genus: ↓*Streptophyta*	No change	[106]

**Table 3:** Interactions between fungal and bacterial communities. Note that ↑ is increased and ↓ decreased.

Fungi	Host	Type of Sample	Site of Sample	Bacterial Microbiota Method	Bacterial Microbiota Change	Diversity Profile	Reference
*Candida albicans*				Culture	*Clostridium difficile*↑	Not evaluated	[124]
				Culture; qPCR	Family: Enterobacteriaceae↑	Not evaluated	[125]
				Culture	*Bacteroides fragilis*↑, *Bacteroides vulgatus*↑	Not evaluated	[126]
	*Clostridium difficile* in C57BL/6 mice	Distal Cecum, contents		High-throughput sequencing of 16S rRNA amplicons (Illimuna)	Phylum: Verrucomicrobia↑, Proteobacteria↑, Actinobacteria↑, Firmicutes↑, Bacteroidetes↑ Family: Comamonadaceae↑, Erysipelotrichacea↑, S24-7↑ Genus: *Akkermansia* sp.↑, *Sutterella* sp.↑, *Bifidobacterium* sp.↑, *Adlercreutzia* sp.↑	↓alpha diversity	[127]
	Mouse model of *Clostridium difficile*	Faeces	Colon	Culture	*Clostridium difficile*↑	Not evaluated	[128]
*Debaryomyces hansenii*↑ *Candida* spp.↓, and *Saccharomyces* spp.↓	Obese children	Faeces		Denaturing gradient gel electrophoresis (DGGE) qPCR	Species: *Akkermansia muciniphila*↓, *Faecalibacterium prausnitzii*↓	↑alpha diversity	[114]
*Ganoderma lucidum mycelium*	High-Fat Diet (HFD)-fed Mice Chow mice	Faeces	Caecal	Pyrosequencing of bacterial 16S rRNA	Phylum: Firmicutes-to-Bacteroidetes ratios↓, Proteobacteria↓ Species: *Parabacteroides goldsteinii*↑, *Bacteroides*↑, *Anaerotruncus colihominis*↑, *Roseburia hominis*↑, *Clostridium*↑, *Clostridium methylpentosum*↑, *Clostridium* XIVa and XVIII↑, *Eubacterium coprostanoligenes*↑	↑alpha diversity	[118]
*Macrorhabdus ornithogaster*	Canaries (*Serinus canaria domestica*)	Faeces		PCR-DGGE, High-throughput sequencing of 16S rRNA amplicons (Illimuna)	Phylum: Acidobacteria↑, Actinobacteria↑, Cyanobacteria↑, Planctomycetes↑ Family: Lachnospiraceae↓, Enterobacteriaceae↓ Genus: *Lactobacillus*↑, *Streptococcus*↑, *Clostridium*↓, *Lactococcus*↓, *Pseudomonas*↓, *Acinetobacter*↓, *Weissella*↓, *Propionibacterium*↓ Species: *Candidatus Arthromitus*↑	↓alpha diversity	[129]
Mushroom (*Agaricus bisporus*)	Pigs	Faeces, Proximal colon contents		High-throughput sequencing of 16S rRNA amplicons (Ion Torrent)	Family*:* Lachnospiraceae↑*,* Ruminococcaceae↑ Order: Clostridiales↑	No change	[130]
*Mucor circinelloides*	BALB/C mice	Faeces		High-throughput sequencing of 16S rRNA amplicons (Illimuna)	Genus: *Bacteroides*↑ Species: *Akkermansia muciniphila*↑	↑alpha diversity	[131]
*Mucor velutinosus*	Old man with Onychomycosis and acute myelogenous leukemia	Oral Stool		High-throughput sequencing of 16S rRNA amplicons (Illimuna)	staphylococci↑	↓alpha diversity	[119]
*Malassezia*↓, *Saccharomyces* sp.↓	Children with Hirschsprung disease	Faeces		High-throughput sequencing of 16S rRNA amplicons (Illimuna; Ion Torrent)	Phylum: Firmicutes↓, Verrucomicrobia↓, Bacteroidetes↓, Proteobacteria↓	↓alpha diversity	[117]
*Nosema ceranae*	Adult workers Honeybees (*Apis mellifera*)		Hindguts	qPCR	*Lactobacillus* spp.↓ and *Bifidobacterium* spp.↓, *Snodgrassella alvi*↑, *Gilliamella apicola*↑	Not evaluated	[132]
*Paranosema locustae*	*Locusta* *migratoria* *manilensis*	Faeces	Hindgut	High-throughput Pyrosequencing of 16S rRNA amplicons (454)	Genus: *Citrobacter*↑, *Lactococcus*↑, *Raoultella*↑ Species: *Corynebacterium* sp. WA7↓, *Raoultella terrigena*↓	↓alpha diversity	[122]
*Sordariomycetes* *Eurotiomycetes* *Dothideomycetes* *Leotiomycetes*	*Pantala flavescens*	Fresh mycelia	Larvae	Culture; High-throughput sequencing of 16S rRNA	Phylum: Proteobacteria↑, Firmicutes↑ Genus: *Sphingomonas*, *Methylobacterium*, *Burkholderia*, *Pantoea*, *Enterobacter*↑, *Leclercia*, and *Serratia*, *Oceanobacillus* Species: *Leclercia* sp., *Oceanobacillus oncorhynchi*, *Methylobacterium extorquens*	Not evaluated	[123]
*Saccharomyces* *boulardii*	Premature infants	Faeces		High-throughput sequencing of 16S rRNA amplicons (Ion Torrent)	Phylum: Proteobacteria↓, Bacteroidetes↓, Actinobacteria↓ Genus: *Escherichia*↑, *Enterococcus*↓, *Veilonella*↑, *Clostridium*↑, *Bifidobacteriu*↑	↑alpha diversity	[115]
	Hamster hypercholesterolemic model			High-throughput Sequencing of 16S rRNA amplicons (Illumina)	Phylum: Firmicutes↓, Tenericutes↓, *TM7*↓, Proteobacteria↑, Lentispharerae↑, unknown phyla↑ Genus: *Allobaculum*↑, CF231↑	No difference	[133]
*Saccharomyces cerevisiae*	Male BALB/c mice		Caecum	Culture	Family: *Enterobacteriaceae*↓	Not evaluated	[134]
	Rats	Faeces		Terminal restriction Fragment length Polymorphism (T-RFLP) analysis	Genus: *Bacteroides*↑, *Fusobacterium*↑, *Bifidobacterium*↑, *Lactobacilli*↑, *Enterococcus*↑	↑alpha diversity	[112]
			Colon	Cuture High-throughput Sequencing of 16S rRNA amplicons (Illumina)	Genus: *Bifidobacterium*↓, *Allobaculum*↓, *Acetanaerobacterium*↑, *Bacteroides*↑, *Eubacterium*↑, *Johnsonella*↑, *Lactococcus*↑, *Oscillospira*↑, *Roseburia*↑, *Vallitalea*↑ Species: *Staphylococcus* spp.↑, haemolytic bacteria↑	Not evaluated	[135]
EpiCor fermentate (dried yeast fermentate made using *Saccharomy* *cescerevisiae*)	Healthy volunteers (symptoms of gastrointestinal discomfort and reduced bowel movements)	Faeces		High-throughput Sequencing of 16S rRNA amplicons (Illumina and 454)	Phylum: Firmicutes↓, Bacteroidetes↑ Family: Bacteroidaceae↑, Porphyromonadaceae↑, Prevotellaceae↑ Genus: *Propionibacterium*↑, *Paraprevotella*↑, *Oscillibacter*↑, *Barnesiella*↑, *Prevotella*↑, *Akkermansia*↑, *Odoribacte*↑, *Anaerostipes*↑, *Blautia*↓, *Roseburia*↓ Specis: *Akkermansia muciniphila*↑	Community evenness↑	[113]

## References

[1] Virgin H.W. (2014). The virome in mammalian physiology and disease. Cell.

[2] Boyer M., Madoui M.-A., Gimenez G., Scola B.L., Raoult D. (2010). Phylogenetic and Phyletic Studies of Informational Genes in Genomes Highlight Existence of a 4th Domain of Life Including Giant Viruses. PLoS ONE.

[3] Raoult D. (2013). TRUC or the Need for a New Microbial Classification. INT.

[4] Lynch S.V., Pedersen O. (2016). The Human Intestinal Microbiome in Health and Disease. N. Engl. J. Med..

[5] Yatsunenko T., Rey F.E., Manary M.J., Trehan I., Dominguez-Bello M.G., Contreras M., Magris M., Hidalgo G., Baldassano R.N., Anokhin A.P. (2012). Human gut microbiome viewed across age and geography. Nature.

[6] Wu G.D., Chen J., Hoffmann C., Bittinger K., Chen Y.-Y., Keilbaugh S.A., Bewtra M., Knights D., Walters W.A., Knight R. (2011). Linking Long-Term Dietary Patterns with Gut Microbial Enterotypes. Science.

[7] David L.A., Maurice C.F., Carmody R.N., Gootenberg D.B., Button J.E., Wolfe B.E., Ling A.V., Devlin A.S., Varma Y., Fischbach M.A. (2014). Diet rapidly and reproducibly alters the human gut microbiome. Nature.

[8] Xu Z., Knight R. (2015). Dietary effects on human gut microbiome diversity. Br. J. Nutr..

[9] Falony G., Joossens M., Vieira-Silva S., Wang J., Darzi Y., Faust K., Kurilshikov A., Bonder M.J., Valles-Colomer M., Vandeputte D. (2016). Population-level analysis of gut microbiome variation. Science.

[10] Vandeputte D., Falony G., Vieira-Silva S., Tito R.Y., Joossens M., Raes J. (2016). Stool consistency is strongly associated with gut microbiota richness and composition, enterotypes and bacterial growth rates. Gut.

[11] Ley R.E., Turnbaugh P.J., Klein S., Gordon J.I. (2006). Human gut microbes associated with obesity. Nature.

[12] Zhernakova A., Kurilshikov A., Bonder M.J., Tigchelaar E.F., Schirmer M., Vatanen T., Mujagic Z., Vila A.V., Falony G., Vieira-Silva S. (2016). Population-based metagenomics analysis reveals markers for gut microbiome composition and diversity. Science.

[13] Francino M.P. (2016). Antibiotics and the Human Gut Microbiome: Dysbioses and Accumulation of Resistances. Front. Microbiol..

[14] Smits S.A., Leach J., Sonnenburg E.D., Gonzalez C.G., Lichtman J.S., Reid G., Knight R., Manjurano A., Changalucha J., Elias J.E. (2017). Seasonal cycling in the gut microbiome of the Hadza hunter-gatherers of Tanzania. Science.

[15] Liu K., Liu Y., Jiao N., Xu B., Gu Z., Xing T., Xiong J. (2017). Bacterial community composition and diversity in Kalakuli, an alpine glacial-fed lake in Muztagh Ata of the westernmost Tibetan Plateau. FEMS Microbiol. Ecol..

[16] Goodrich J.K., Waters J.L., Poole A.C., Sutter J.L., Koren O., Blekhman R., Beaumont M., Van Treuren W., Knight R., Bell J.T. (2014). Human Genetics Shape the Gut Microbiome. Cell.

[17] Blekhman R., Goodrich J.K., Huang K., Sun Q., Bukowski R., Bell J.T., Spector T.D., Keinan A., Ley R.E., Gevers D. (2015). Host genetic variation impacts microbiome composition across human body sites. Genome Biol..

[18] Goodrich J.K., Davenport E.R., Beaumont M., Jackson M.A., Knight R., Ober C., Spector T.D., Bell J.T., Clark A.G., Ley R.E. (2016). Genetic Determinants of the Gut Microbiome in UK Twins. Cell Host Microbe.

[19] Frank D.N., Pace N.R. (2008). Gastrointestinal microbiology enters the metagenomics era. Curr. Opin. Gastroenterol..

[20] Li J., Jia H., Cai X., Zhong H., Feng Q., Sunagawa S., Arumugam M., Kultima J.R., Prifti E., Nielsen T. (2014). An integrated catalog of reference genes in the human gut microbiome. Nat. Biotechnol..

[21] Porter J.R. (1976). Antony van Leeuwenhoekl: Tercentenary of His Discovery of Bacteria. Bacteriol. Rev..

[22] Lagier J.-C., Edouard S., Pagnier I., Mediannikov O., Drancourt M., Raoult D. (2015). Current and Past Strategies for Bacterial Culture in Clinical Microbiology. Clin. Microbiol. Rev..

[23] Fournier P.-E., Drancourt M., Colson P., Rolain J.-M., Scola B.L., Raoult D. (2013). Modern clinical microbiology: New challenges and solutions. Nat. Rev. Microbiol..

[24] Dave M., Purohit T., Razonable R., Loftus E.V. (2014). Opportunistic Infections Due to Inflammatory Bowel Disease Therapy. Inflamm. Bowel Dis..

[25] Hugon P., Dufour J.-C., Colson P., Fournier P.-E., Sallah K., Raoult D. (2015). A comprehensive repertoire of prokaryotic species identified in human beings. Lancet Infect. Dis..

[26] Lagier J.-C., Drancourt M., Charrel R., Bittar F., La Scola B., Ranque S., Raoult D. (2017). Many More Microbes in Humans: Enlarging the Microbiome Repertoire. Clin. Infect. Dis..

[27] O’Brien Andersen L., Karim A.B., Roager H.M., Vigsnæs L.K., Krogfelt K.A., Licht T.R., Stensvold C.R. (2016). Associations between common intestinal parasites and bacteria in humans as revealed by qPCR. Eur. J. Clin. Microbiol. Infect. Dis..

[28] Iebba V., Santangelo F., Totino V., Pantanella F., Monsia A., Di Cristanziano V., Di Cave D., Schippa S., Berrilli F., D’Alfonso R. (2016). Gut microbiota related to Giardia duodenalis, Entamoeba spp. and Blastocystis hominis infections in humans from Côte d’Ivoire. J. Infect. Dev. Ctries..

[29] Audebert C., Even G., Cian A., Loywick A., Merlin S., Viscogliosi E., Chabé M., Blastocystis Investigation Group (2016). Colonization with the enteric protozoa Blastocystis is associated with increased diversity of human gut bacterial microbiota. Sci. Rep..

[30] Beatty J.K., Akierman S.V., Motta J.-P., Muise S., Workentine M.L., Harrison J.J., Bhargava A., Beck P.L., Rioux K.P., McKnight G.W. (2017). Giardia duodenalis induces pathogenic dysbiosis of human intestinal microbiota biofilms. Int. J. Parasitol..

[31] Gilchrist C.A., Petri S.E., Schneider B.N., Reichman D.J., Jiang N., Begum S., Watanabe K., Jansen C.S., Elliott K.P., Burgess S.L. (2016). Role of the Gut Microbiota of Children in Diarrhea Due to the Protozoan Parasite *Entamoeba histolytica*. J. Infect. Dis..

[32] Yooseph S., Kirkness E.F., Tran T.M., Harkins D.M., Jones M.B., Torralba M.G., O’Connell E., Nutman T.B., Doumbo S., Doumbo O.K. (2015). Stool microbiota composition is associated with the prospective risk of Plasmodium falciparum infection. BMC Genom..

[33] Villarino N.F., LeCleir G.R., Denny J.E., Dearth S.P., Harding C.L., Sloan S.S., Gribble J.L., Campagna S.R., Wilhelm S.W., Schmidt N.W. (2016). Composition of the gut microbiota modulates the severity of malaria. Proc. Natl. Acad. Sci. USA.

[34] Yang C.-A., Liang C., Lin C.-L., Hsiao C.-T., Peng C.-T., Lin H.-C., Chang J.-G. (2017). Impact of Enterobius vermicularis infection and mebendazole treatment on intestinal microbiota and host immune response. PLoS Negl. Trop. Dis..

[35] Holm J.B., Sorobetea D., Kiilerich P., Ramayo-Caldas Y., Estellé J., Ma T., Madsen L., Kristiansen K., Svensson-Frej M. (2015). Chronic Trichuris muris Infection Decreases Diversity of the Intestinal Microbiota and Concomitantly Increases the Abundance of Lactobacilli. PLoS ONE.

[36] Jenkins T.P., Rathnayaka Y., Perera P.K., Peachey L.E., Nolan M.J., Krause L., Rajakaruna R.S., Cantacessi C. (2017). Infections by human gastrointestinal helminths are associated with changes in faecal microbiota diversity and composition. PLoS ONE.

[37] Lamendella R., Wright J.R., Hackman J., McLimans C., Toole D.R., Bernard Rubio W., Drucker R., Wong H.T., Sabey K., Hegarty J.P. (2018). Antibiotic Treatments for *Clostridium difficile* Infection Are Associated with Distinct Bacterial and Fungal Community Structures. mSphere.

[38] Liguori G., Lamas B., Richard M.L., Brandi G., da Costa G., Hoffmann T.W., Di Simone M.P., Calabrese C., Poggioli G., Langella P. (2016). Fungal Dysbiosis in Mucosa-associated Microbiota of Crohn’s Disease Patients. J. Crohn’s Colitis.

[39] Eckburg P.B., Bik E.M., Bernstein C.N., Purdom E., Dethlefsen L., Sargent M., Gill S.R., Nelson K.E., Relman D.A. (2005). Diversity of the Human Intestinal Microbial Flora. Science.

[40] Costa M., Weese J.S. (2019). Methods and basic concepts for microbiota assessment. Vet. J..

[41] Lagier J.-C., Hugon P., Khelaifia S., Fournier P.-E., La Scola B., Raoult D. (2015). The Rebirth of Culture in Microbiology through the Example of Culturomics to Study Human Gut Microbiota. Clin. Microbiol. Rev..

[42] Muyzer G., Smalla K. (1998). Application of denaturing gradient gel electrophoresis (DGGE) and temperature gradient gel electrophoresis (TGGE) in microbial ecology. Antonie Van Leeuwenhoek.

[43] Green S.J., Leigh M.B., Neufeld J.D., McGenity T.J., Timmis K.N., Nogales B. (2017). Denaturing Gradient Gel Electrophoresis (DGGE) for Microbial Community Analysis. Hydrocarbon and Lipid Microbiology Protocols: Microbial Quantitation, Community Profiling and Array Approaches.

[44] Meroth C.B., Walter J., Hertel C., Brandt M.J., Hammes W.P. (2003). Monitoring the bacterial population dynamics in sourdough fermentation processes by using PCR-denaturing gradient gel electrophoresis. Appl. Environ. Microbiol..

[45] de Souza F.A., Kowalchuk G.A., Leeflang P., van Veen J.A., Smit E. (2004). PCR-Denaturing Gradient Gel Electrophoresis Profiling of Inter- and Intraspecies 18S rRNA Gene Sequence Heterogeneity Is an Accurate and Sensitive Method To Assess Species Diversity of Arbuscular Mycorrhizal Fungi of the Genus Gigaspora. Appl. Environ. Microbiol..

[46] Ariefdjohan M.W., Savaiano D.A., Nakatsu C.H. (2010). Comparison of DNA extraction kits for PCR-DGGE analysis of human intestinal microbial communities from fecal specimens. Nutr. J..

[47] Handschur M., Pinar G., Gallist B., Lubitz W., Haslberger A.G. (2005). Culture free DGGE and cloning based monitoring of changes in bacterial communities of salad due to processing. Food Chem. Toxicol..

[48] Riemann L., Winding A. (2001). Community Dynamics of Free-living and Particle-associated Bacterial Assemblages during a Freshwater Phytoplankton Bloom. Microb. Ecol..

[49] Heuer H., Hartung K., Wieland G., Kramer I., Smalla K. (1999). Polynucleotide Probes That Target a Hypervariable Region of 16S rRNA Genes to Identify Bacterial Isolates Corresponding to Bands of Community Fingerprints. Appl. Environ. Microbiol..

[50] Theron J., Cloete T.E. (2000). Molecular Techniques for Determining Microbial Diversity and Community Structure in Natural Environments. Crit. Rev. Microbiol..

[51] Wintzingerode F.V., Göbel U.B., Stackebrandt E. (1997). Determination of microbial diversity in environmental samples: Pitfalls of PCR-based rRNA analysis. FEMS Microbiol. Rev..

[52] Gelsomino A., Keijzer-Wolters A.C., Cacco G., van Elsas J.D. (1999). Assessment of bacterial community structure in soil by polymerase chain reaction and denaturing gradient gel electrophoresis. J. Microbiol. Methods.

[53] Sanschagrin S., Yergeau E. (2014). Next-generation Sequencing of 16S Ribosomal RNA Gene Amplicons. J. Vis. Exp..

[54] Shendure J., Ji H. (2008). Next-generation DNA sequencing. Nat. Biotechnol..

[55] Blazej R.G., Kumaresan P., Mathies R.A. (2006). Microfabricated bioprocessor for integrated nanoliter-scale Sanger DNA sequencing. Proc. Natl. Acad. Sci. USA.

[56] Gresham D., Dunham M.J., Botstein D. (2008). Comparing whole genomes using DNA microarrays. Nat. Rev. Genet..

[57] Soni G.V., Meller A. (2007). Progress toward Ultrafast DNA Sequencing Using Solid-State Nanopores. Clin. Chem..

[58] Shendure J. (2005). Accurate Multiplex Polony Sequencing of an Evolved Bacterial Genome. Science.

[59] Healy K. (2007). Nanopore-based single-molecule DNA analysis. Nanomedicine.

[60] Salipante S.J., Kawashima T., Rosenthal C., Hoogestraat D.R., Cummings L.A., Sengupta D.J., Harkins T.T., Cookson B.T., Hoffman N.G. (2014). Performance Comparison of Illumina and Ion Torrent Next-Generation Sequencing Platforms for 16S rRNA-Based Bacterial Community Profiling. Appl. Environ. Microbiol..

[61] Kuczynski J., Lauber C.L., Walters W.A., Parfrey L.W., Clemente J.C., Gevers D., Knight R. (2011). Experimental and analytical tools for studying the human microbiome. Nat. Rev. Genet..

[62] Luo C., Rodriguez-R L.M., Konstantinidis K.T. (2013). A User’s Guide to Quantitative and Comparative Analysis of Metagenomic Datasets. Methods in Enzymology.

[63] Luo C., Rodriguez-R L.M., Konstantinidis K.T. (2014). MyTaxa: An advanced taxonomic classifier for genomic and metagenomic sequences. Nucleic Acids Res..

[64] Sims D., Sudbery I., Ilott N.E., Heger A., Ponting C.P. (2014). Sequencing depth and coverage: Key considerations in genomic analyses. Nat. Rev. Genet..

[65] Ranjan R., Rani A., Metwally A., McGee H.S., Perkins D.L. (2016). Analysis of the microbiome: Advantages of whole genome shotgun versus 16S amplicon sequencing. Biochem. Biophys. Res. Commun..

[66] van Nimwegen K.J.M., van Soest R.A., Veltman J.A., Nelen M.R., van der Wilt G.J., Vissers L.E.L.M., Grutters J.P.C. (2016). Is the $1000 Genome as Near as We Think? A Cost Analysis of Next-Generation Sequencing. Clin. Chem..

[67] Marizzoni M., Gurry T., Provasi S., Greub G., Lopizzo N., Ribaldi F., Festari C., Mazzelli M., Mombelli E., Salvatore M. (2020). Comparison of Bioinformatics Pipelines and Operating Systems for the Analyses of 16S rRNA Gene Amplicon Sequences in Human Fecal Samples. Front. Microbiol..

[68] Corless C.E., Guiver M., Borrow R., Edwards-Jones V., Kaczmarski E.B., Fox A.J. (2000). Contamination and Sensitivity Issues with a Real-Time Universal 16S rRNA PCR. J. Clin. Microbiol..

[69] Pereira-Marques J., Hout A., Ferreira R.M., Weber M., Pinto-Ribeiro I., van Doorn L.-J., Knetsch C.W., Figueiredo C. (2019). Impact of Host DNA and Sequencing Depth on the Taxonomic Resolution of Whole Metagenome Sequencing for Microbiome Analysis. Front. Microbiol..

[70] Poretsky R., Rodriguez-R L.M., Luo C., Tsementzi D., Konstantinidis K.T. (2014). Strengths and Limitations of 16S rRNA Gene Amplicon Sequencing in Revealing Temporal Microbial Community Dynamics. PLoS ONE.

[71] Jones M.B., Highlander S.K., Anderson E.L., Li W., Dayrit M., Klitgord N., Fabani M.M., Seguritan V., Green J., Pride D.T. (2015). Library preparation methodology can influence genomic and functional predictions in human microbiome research. Proc. Natl. Acad. Sci. USA.

[72] Chen K., Pachter L. (2005). Bioinformatics for Whole-Genome Shotgun Sequencing of Microbial Communities. PLoS Comput. Biol..

[73] Ras R., Huynh K., Desoky E., Badawy A., Widmer G. (2015). Perturbation of the intestinal microbiota of mice infected with Cryptosporidium parvum. Int. J. Parasitol..

[74] Yildiz S., Doğan İ., Doğruman-Al F., Nalbantoğlu U., Üstek D., Sarzhanov F., Yildirim S. (2016). Association of Enteric Protist Blastocystis spp. and Gut Microbiota with Hepatic Encephalopathy. J. Gastrointest. Liver Dis..

[75] Morton E.R., Lynch J., Froment A., Lafosse S., Heyer E., Przeworski M., Blekhman R., Ségurel L. (2015). Variation in Rural African Gut Microbiota Is Strongly Correlated with Colonization by Entamoeba and Subsistence. PLoS Genet..

[76] Kelly P.H., Bahr S.M., Serafim T.D., Ajami N.J., Petrosino J.F., Meneses C., Kirby J.R., Valenzuela J.G., Kamhawi S., Wilson M.E. (2017). The Gut Microbiome of the Vector Lutzomyia longipalpis Is Essential for Survival of Leishmania infantum. MBio.

[77] Heimesaat M.M., Dunay I.R., Alutis M., Fischer A., Möhle L., Göbel U.B., Kühl A.A., Bereswill S. (2014). Nucleotide-Oligomerization-Domain-2 Affects Commensal Gut Microbiota Composition and Intracerebral Immunopathology in Acute Toxoplasma gondii Induced Murine Ileitis. PLoS ONE.

[78] Brotman R.M., Bradford L.L., Conrad M., Gajer P., Ault K., Peralta L., Forney L.J., Carlton J.M., Abdo Z., Ravel J. (2012). Association between Trichomonas vaginalis and vaginal bacterial community composition among reproductive-age women. Sex. Transm. Dis..

[79] Šlapeta J., Dowd S.E., Alanazi A.D., Westman M.E., Brown G.K. (2015). Differences in the faecal microbiome of non-diarrhoeic clinically healthy dogs and cats associated with Giardia duodenalis infection: Impact of hookworms and coccidia. Int. J. Parasitol..

[80] Kodio A., Coulibaly D., Koné A.K., Konaté S., Doumbo S., Guindo A., Bittar F., Gouriet F., Raoult D., Thera M.A. (2019). Blastocystis Colonization Is Associated with Increased Diversity and Altered Gut Bacterial Communities in Healthy Malian Children. Microorganisms.

[81] Oliveira B.C.M., Widmer G. (2018). Probiotic Product Enhances Susceptibility of Mice to Cryptosporidiosis. Appl. Environ. Microbiol..

[82] Barash N.R., Maloney J.G., Singer S.M., Dawson S.C. (2017). Giardia Alters Commensal Microbial Diversity throughout the Murine Gut. Infect. Immun..

[83] Hatter J.A., Kouche Y.M., Melchor S.J., Ng K., Bouley D.M., Boothroyd J.C., Ewald S.E. (2018). Toxoplasma gondii infection triggers chronic cachexia and sustained commensal dysbiosis in mice. PLoS ONE.

[84] Perez P.F., Minnaard J., Rouvet M., Knabenhans C., Brassart D., De Antoni G.L., Schiffrin E.J. (2001). Inhibition of Giardia intestinalis by Extracellular Factors from Lactobacilli: An In Vitro Study. Appl. Environ. Microbiol..

[85] Alamer E., Carpio V.H., Ibitokou S.A., Kirtley M.L., Phoenix I.R., Opata M.M., Wilson K.D., Cong Y., Dann S.M., Chopra A.K. (2019). Dissemination of non-typhoidal Salmonella during Plasmodium chabaudi infection affects anti-malarial immunity. Parasitol. Res..

[86] Lappan R., Classon C., Kumar S., Singh O.P., de Almeida R.V., Chakravarty J., Kumari P., Kansal S., Sundar S., Blackwell J.M. (2019). Meta-taxonomic analysis of prokaryotic and eukaryotic gut flora in stool samples from visceral leishmaniasis cases and endemic controls in Bihar State India. PLoS Negl. Trop. Dis..

[87] Wu S., Li R.W., Li W., Beshah E., Dawson H.D., Urban J.F. (2012). Worm Burden-Dependent Disruption of the Porcine Colon Microbiota by Trichuris suis Infection. PLoS ONE.

[88] Li R.W., Wu S., Li W., Navarro K., Couch R.D., Hill D., Urban J.F. (2012). Alterations in the Porcine Colon Microbiota Induced by the Gastrointestinal Nematode Trichuris suis. Infect. Immun..

[89] Cooper P., Walker A.W., Reyes J., Chico M., Salter S.J., Vaca M., Parkhill J. (2013). Patent Human Infections with the Whipworm, Trichuris trichiura, Are Not Associated with Alterations in the Faecal Microbiota. PLoS ONE.

[90] Lee S.C., Tang M.S., Lim Y.A.L., Choy S.H., Kurtz Z.D., Cox L.M., Gundra U.M., Cho I., Bonneau R., Blaser M.J. (2014). Helminth Colonization Is Associated with Increased Diversity of the Gut Microbiota. PLoS Negl. Trop. Dis..

[91] Rosa B.A., Supali T., Gankpala L., Djuardi Y., Sartono E., Zhou Y., Fischer K., Martin J., Tyagi R., Bolay F.K. (2018). Differential human gut microbiome assemblages during soil-transmitted helminth infections in Indonesia and Liberia. Microbiome.

[92] Houlden A., Hayes K.S., Bancroft A.J., Worthington J.J., Wang P., Grencis R.K., Roberts I.S. (2015). Chronic Trichuris muris Infection in C57BL/6 Mice Causes Significant Changes in Host Microbiota and Metabolome: Effects Reversed by Pathogen Clearance. PLoS ONE.

[93] Williams A.R., Krych L., Ahmad H.F., Nejsum P., Skovgaard K., Nielsen D.S., Thamsborg S.M. (2017). A polyphenol-enriched diet and Ascaris suum infection modulate mucosal immune responses and gut microbiota composition in pigs. PLoS ONE.

[94] Wegener Parfrey L., Jirků M., Šíma R., Jalovecká M., Sak B., Grigore K., Jirků Pomajbíková K. (2017). A benign helminth alters the host immune system and the gut microbiota in a rat model system. PLoS ONE.

[95] McKenney E.A., Williamson L., Yoder A.D., Rawls J.F., Bilbo S.D., Parker W. (2015). Alteration of the rat cecal microbiome during colonization with the helminth *Hymenolepis diminuta*. Gut Microbes.

[96] Giacomin P., Zakrzewski M., Croese J., Su X., Sotillo J., McCann L., Navarro S., Mitreva M., Krause L., Loukas A. (2015). Experimental hookworm infection and escalating gluten challenges are associated with increased microbial richness in celiac subjects. Sci. Rep..

[97] Vicente C.S.L., Ozawa S., Hasegawa K. (2016). Composition of the Cockroach Gut Microbiome in the Presence of Parasitic Nematodes. Microbes Environ..

[98] El-Ashram S., Suo X. (2017). Exploring the microbial community (microflora) associated with ovine Haemonchus contortus (macroflora) field strains. Sci. Rep..

[99] Kay G.L., Millard A., Sergeant M.J., Midzi N., Gwisai R., Mduluza T., Ivens A., Nausch N., Mutapi F., Pallen M. (2015). Differences in the Faecal Microbiome in Schistosoma haematobium Infected Children vs. Uninfected Children. PLoS Negl. Trop. Dis..

[100] Peachey L.E., Molena R.A., Jenkins T.P., Di Cesare A., Traversa D., Hodgkinson J.E., Cantacessi C. (2018). The relationships between faecal egg counts and gut microbial composition in UK Thoroughbreds infected by cyathostomins. Int. J. Parasitol..

[101] Rausch S., Held J., Fischer A., Heimesaat M.M., Kühl A.A., Bereswill S., Hartmann S. (2013). Small Intestinal Nematode Infection of Mice Is Associated with Increased Enterobacterial Loads alongside the Intestinal Tract. PLoS ONE.

[102] Kreisinger J., Bastien G., Hauffe H.C., Marchesi J., Perkins S.E. (2015). Interactions between multiple helminths and the gut microbiota in wild rodents. Philos. Trans. R. Soc. Lond. B Biol. Sci..

[103] Cattadori I.M., Sebastian A., Hao H., Katani R., Albert I., Eilertson K.E., Kapur V., Pathak A., Mitchell S. (2016). Impact of Helminth Infections and Nutritional Constraints on the Small Intestine Microbiota. PLoS ONE.

[104] Duarte A.M., Jenkins T.P., Latrofa M.S., Giannelli A., Papadopoulos E., de Carvalho L.M., Nolan M.J., Otranto D., Cantacessi C. (2016). Helminth infections and gut microbiota—A feline perspective. Parasites Vectors.

[105] Schneeberger P.H.H., Coulibaly J.T., Panic G., Daubenberger C., Gueuning M., Frey J.E., Keiser J. (2018). Investigations on the interplays between Schistosoma mansoni, praziquantel and the gut microbiome. Parasit Vectors.

[106] Broadhurst M.J., Ardeshir A., Kanwar B., Mirpuri J., Gundra U.M., Leung J.M., Wiens K.E., Vujkovic-Cvijin I., Kim C.C., Yarovinsky F. (2012). Therapeutic Helminth Infection of Macaques with Idiopathic Chronic Diarrhea Alters the Inflammatory Signature and Mucosal Microbiota of the Colon. PLoS Pathog..

[107] Walk S.T., Blum A.M., Ewing S.A.-S., Weinstock J.V., Young V.B. (2010). Alteration of the murine gut microbiota during infection with the parasitic helminth, Heligmosomoides polygyrus. Inflamm. Bowel Dis..

[108] Shimokawa C., Obi S., Shibata M., Olia A., Imai T., Suzue K., Hisaeda H. (2019). Suppression of Obesity by an Intestinal Helminth through Interactions with Intestinal Microbiota. Infect. Immun..

[109] Jenkins T.P., Peachey L.E., Ajami N.J., MacDonald A.S., Hsieh M.H., Brindley P.J., Cantacessi C., Rinaldi G. (2018). Schistosoma mansoni infection is associated with quantitative and qualitative modifications of the mammalian intestinal microbiota. Sci. Rep..

[110] Williamson L.L., McKenney E.A., Holzknecht Z.E., Belliveau C., Rawls J.F., Poulton S., Parker W., Bilbo S.D. (2016). Got worms? Perinatal exposure to helminths prevents persistent immune sensitization and cognitive dysfunction induced by early-life infection. Brain Behav. Immun..

[111] Wang Y., Liu F., Urban J.F., Paerewijck O., Geldhof P., Li R.W. (2019). Ascaris suum infection was associated with a worm-independent reduction in microbial diversity and altered metabolic potential in the porcine gut microbiome. Int. J. Parasitol..

[112] Li S., Sha Z., Wang X., Bu Z., Wang L., Guan X., Lang X., Wang X. (2017). Yeast Surface Display of Escherichia coli Enterotoxin and Its Effects of Intestinal Microflora and Mucosal Immunity. Curr. Microbiol..

[113] Pinheiro I., Robinson L., Verhelst A., Marzorati M., Winkens B., den Abbeele P.V., Possemiers S. (2017). A yeast fermentate improves gastrointestinal discomfort and constipation by modulation of the gut microbiome: Results from a randomized double-blind placebo-controlled pilot trial. BMC Complement. Altern. Med..

[114] Borgo F., Verduci E., Riva A., Lassandro C., Riva E., Morace G., Borghi E. (2017). Relative Abundance in Bacterial and Fungal Gut Microbes in Obese Children: A Case Control Study. Child. Obes..

[115] Zeber-Lubecka N., Kulecka M., Ambrozkiewicz F., Paziewska A., Lechowicz M., Konopka E., Majewska U., Borszewska-Kornacka M., Mikula M., Cukrowska B. (2016). Effect of Saccharomyces boulardii and Mode of Delivery on the Early Development of the Gut Microbial Community in Preterm Infants. PLoS ONE.

[116] Sokol H., Leducq V., Aschard H., Pham H.-P., Jegou S., Landman C., Cohen D., Liguori G., Bourrier A., Nion-Larmurier I. (2017). Fungal microbiota dysbiosis in IBD. Gut.

[117] Frykman P.K., Nordenskjöld A., Kawaguchi A., Hui T.T., Granström A.L., Cheng Z., Tang J., Underhill D.M., Iliev I., Funari V.A. (2015). Characterization of Bacterial and Fungal Microbiome in Children with Hirschsprung Disease with and without a History of Enterocolitis: A Multicenter Study. PLoS ONE.

[118] Chang C.-J., Lin C.-S., Lu C.-C., Martel J., Ko Y.-F., Ojcius D.M., Tseng S.-F., Wu T.-R., Chen Y.-Y.M., Young J.D. (2015). Ganoderma lucidum reduces obesity in mice by modulating the composition of the gut microbiota. Nat. Commun..

[119] Shelburne S.A., Ajami N.J., Chibucos M.C., Beird H.C., Tarrand J., Galloway-Peña J., Albert N., Chemaly R.F., Ghantoji S.S., Marsh L. (2015). Implementation of a Pan-Genomic Approach to Investigate Holobiont-Infecting Microbe Interaction: A Case Report of a Leukemic Patient with Invasive Mucormycosis. PLoS ONE.

[120] Hoarau G., Mukherjee P.K., Gower-Rousseau C., Hager C., Chandra J., Retuerto M.A., Neut C., Vermeire S., Clemente J., Colombel J.F. (2016). Bacteriome and Mycobiome Interactions Underscore Microbial Dysbiosis in Familial Crohn’s Disease. MBio.

[121] Strati F., Cavalieri D., Albanese D., De Felice C., Donati C., Hayek J., Jousson O., Leoncini S., Pindo M., Renzi D. (2016). Altered gut microbiota in Rett syndrome. Microbiome.

[122] Tan S.-Q., Zhang K.-Q., Chen H.-X., Ge Y., Ji R., Shi W.-P. (2015). The mechanism for microsporidian parasite suppression of the hindgut bacteria of the migratory locust Locusta migratoria manilensis. Sci. Rep..

[123] Shao M.-W., Lu Y.-H., Miao S., Zhang Y., Chen T.-T., Zhang Y.-L. (2015). Diversity, Bacterial Symbionts and Antibacterial Potential of Gut-Associated Fungi Isolated from the Pantala flavescens Larvae in China. PLoS ONE.

[124] van Leeuwen P.T., van der Peet J.M., Bikker F.J., Hoogenkamp M.A., Oliveira Paiva A.M., Kostidis S., Mayboroda O.A., Smits W.K., Krom B.P. (2016). Interspecies Interactions between *Clostridium difficile* and *Candida albicans*. mSphere.

[125] Sovran B., Planchais J., Jegou S., Straube M., Lamas B., Natividad J.M., Agus A., Dupraz L., Glodt J., Da Costa G. (2018). Enterobacteriaceae are essential for the modulation of colitis severity by fungi. Microbiome.

[126] Valentine M., Benadé E., Mouton M., Khan W., Botha A. (2019). Binary interactions between the yeast Candida albicans and two gut-associated Bacteroides species. Microb. Pathog..

[127] Markey L., Shaban L., Green E.R., Lemon K.P., Mecsas J., Kumamoto C.A. (2018). Pre-colonization with the commensal fungus Candida albicans reduces murine susceptibility to Clostridium difficile infection. Gut Microbes.

[128] Panpetch W., Somboonna N., Palasuk M., Hiengrach P., Finkelman M., Tumwasorn S., Leelahavanichkul A. (2019). Oral Candida administration in a Clostridium difficile mouse model worsens disease severity but is attenuated by Bifidobacterium. PLoS ONE.

[129] Robino P., Ferrocino I., Rossi G., Dogliero A., Alessandria V., Grosso L., Galosi L., Tramuta C., Cocolin L., Nebbia P. (2019). Changes in gut bacterial communities in canaries infected by Macrorhabdus ornithogaster. Avian Pathol..

[130] Solano-Aguilar G.I., Jang S., Lakshman S., Gupta R., Beshah E., Sikaroodi M., Vinyard B., Molokin A., Gillevet P.M., Urban J.F. (2018). The Effect of Dietary Mushroom Agaricus bisporus on Intestinal Microbiota Composition and Host Immunological Function. Nutrients.

[131] Mueller K.D., Zhang H., Serrano C.R., Billmyre R.B., Huh E.Y., Wiemann P., Keller N.P., Wang Y., Heitman J., Lee S.C. (2019). Gastrointestinal microbiota alteration induced by Mucor circinelloides in a murine model. J. Microbiol..

[132] Rouzé R., Moné A., Delbac F., Belzunces L., Blot N. (2019). The Honeybee Gut Microbiota Is Altered after Chronic Exposure to Different Families of Insecticides and Infection by Nosema ceranae. Microbes Environ..

[133] Briand F., Sulpice T., Giammarinaro P., Roux X. (2019). Saccharomyces boulardii CNCM I-745 changes lipidemic profile and gut microbiota in a hamster hypercholesterolemic model. Benef. Microbes.

[134] García G., Dogi C., de Moreno de LeBlanc A., Greco C., Cavaglieri L. (2016). Gut-borne Saccharomyces cerevisiae, a promising candidate for the formulation of feed additives, modulates immune system and gut microbiota. Benef. Microbes.

[135] Ducray H.A.G., Globa L., Pustovyy O., Morrison E., Vodyanoy V., Sorokulova I. (2019). Yeast fermentate prebiotic improves intestinal barrier integrity during heat stress by modulation of the gut microbiota in rats. J. Appl. Microbiol..

[136] Matsuo K., Haku A., Bi B., Takahashi H., Kamada N., Yaguchi T., Saijo S., Yoneyama M., Goto Y. (2019). Fecal microbiota transplantation prevents Candida albicans from colonizing the gastrointestinal tract. Microbiol. Immunol..

[137] Charlet R., Pruvost Y., Tumba G., Istel F., Poulain D., Kuchler K., Sendid B., Jawhara S. (2018). Remodeling of the Candida glabrata cell wall in the gastrointestinal tract affects the gut microbiota and the immune response. Sci. Rep..

[138] Wang J., Liu S., Wang H., Xiao S., Li C., Li Y., Liu B. (2019). Xanthophyllomyces dendrorhous-Derived Astaxanthin Regulates Lipid Metabolism and Gut Microbiota in Obese Mice Induced by A High-Fat Diet. Mar. Drugs.

[139] Swidsinski A., Loening-Baucke V., Schulz S., Manowsky J., Verstraelen H., Swidsinski S. (2016). Functional anatomy of the colonic bioreactor: Impact of antibiotics and Saccharomyces boulardii on bacterial composition in human fecal cylinders. Syst. Appl. Microbiol..

[140] Lemoinne S., Kemgang A., Belkacem K.B., Straube M., Jegou S., Corpechot C., Network S.-A.I., Chazouillères O., Housset C., Sokol H. (2020). Fungi participate in the dysbiosis of gut microbiota in patients with primary sclerosing cholangitis. Gut.

[141] Coker O.O., Nakatsu G., Dai R.Z., Wu W.K.K., Wong S.H., Ng S.C., Chan F.K.L., Sung J.J.Y., Yu J. (2019). Enteric fungal microbiota dysbiosis and ecological alterations in colorectal cancer. Gut.

[142] Sam Q.H., Chang M.W., Chai L.Y.A. (2017). The Fungal Mycobiome and Its Interaction with Gut Bacteria in the Host. Int. J. Mol. Sci..

[143] Hoffmann C., Dollive S., Grunberg S., Chen J., Li H., Wu G.D., Lewis J.D., Bushman F.D. (2013). Archaea and Fungi of the Human Gut Microbiome: Correlations with Diet and Bacterial Residents. PLoS ONE.

[144] Mwangi W., de Figueiredo P., Criscitiello M.F. (2016). One Health: Addressing Global Challenges at the Nexus of Human, Animal, and Environmental Health. PLoS Pathog..

[145] Ryu S., Kim B.I., Lim J.-S., Tan C.S., Chun B.C. (2017). One Health Perspectives on Emerging Public Health Threats. J. Prev. Med. Public Health.

